# “Cicada Out of the Shell” Deep Penetration and Blockage of the HSP90 Pathway by ROS‐Responsive Supramolecular Gels to Augment Trimodal Synergistic Therapy

**DOI:** 10.1002/advs.202401214

**Published:** 2024-04-22

**Authors:** Fashun Li, Jianqin Yan, Chen Wei, Yi Zhao, Xiaowen Tang, Long Xu, Bin He, Yong Sun, Jing Chang, Yan Liang

**Affiliations:** ^1^ Department of Pharmaceutics School of Pharmacy Qingdao University Qingdao 266073 China; ^2^ Department of Pharmacy Qingdao Women and Children's Hospital Qingdao 266034 China; ^3^ Department of Recuperation Medicine Qingdao Special Service Sanatorium of PLA Navy Qingdao 266071 China; ^4^ Department of Medicinal Chemistry School of Pharmacy Qingdao University Qingdao 266073 China; ^5^ School of Materials Science and Chemical Engineering Ningbo University Ningbo 315211 China; ^6^ National Engineering Research Center for Biomaterials Sichuan University Chengdu 610064 China; ^7^ College of Marine Life Science Ocean University of China Qingdao 266003 China

**Keywords:** HSP90, multimodal synergistic therapy, penetration, reactive oxygen species‐responsive, supramolecular gels

## Abstract

Deep penetration and downregulation of heat shock protein (HSP) expression in multimodal synergistic therapy are promising approaches for curing cancer in clinical trials. However, free small‐molecule drugs and most drug vehicles have a low delivery efficiency deep into the tumor owing to poor drug penetration and hypoxic conditions at the tumor site. In this study, the objective is to use reactive oxygen species (ROS)‐responsive supramolecular gels co‐loaded with the photosensitizer Zn(II) phthalocyanine tetrasulfonic acid (ZnPCS_4_) and functionalized tetrahedral DNA (TGSAs) (G@P/TGSAs) to enhance deep tissue and cell penetration and block the HSP90 pathway for chemo‐ photodynamic therapy (PDT) ‐ photothermal therapy (PTT) trimodal synergistic therapy. The (G@P/TGSAs) are injected in situ into the tumor to release ZnPCS_4_ and TGSAs under high ROS concentrations originating from both the tumor and PDT. TGSAs penetrate deeply into tumor tissues and augment photothermal therapy by inhibiting the HSP90 pathway. Proteomics show that HSP‐related proteins and molecular chaperones are inhibited/activated, inhibiting the HSP90 pathway. Simultaneously, the TGSA‐regulated apoptotic pathway is activated. In vivo study demonstrates efficient tumor penetration and excellent trimodal synergistic therapy (45% tumor growth inhibition).

## Graphical abstract

Deep penetration and downregulation of heat shock protein expression in multimodal synergistic therapy are promising approaches for curing cancer. “Cicada Out of the Shell” deep penetration and blockage of heat shock protein pathway by reactive oxygen species‐responsive supramolecular gels to augment trimodal synergistic therapy and in vitro or in vivo study demonstrated efficient tumor penetration and excellent trimodal synergistic therapy.

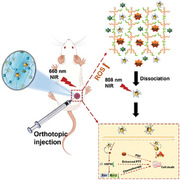

## 1 Introduction

Multimodal synergistic therapies, including bimodal and trimodal therapies based on drug delivery systems, have provided a highly effective synergistic and cancer inhibition approach in clinical trials.^[^ ^1^, ^2^ ^]^ Among these, photodynamic therapy (PDT), photothermal therapy (PTT), and chemotherapy‐based trimodal therapy are particularly promising. In this innovative strategy, PDT utilizes photosensitizers to generate reactive oxygen species (ROS) that efficiently eradicate cancer cells under light irradiation. PTT exploits the heat generated by light‐absorbing materials to kill tumor cells^[^ ^3^, ^4^, ^5^ ^]^ Despite the potential of PTT‐induced heat to enhance nanoparticle uptake by tumor cells and facilitate the release of photosensitizers and antitumor drugs into the cytoplasm,^[^ ^6^ ^]^ there remain several limitations to multimode therapy that hinder the achievement of anti‐tumor effects that meet clinical requirements.

ROS levels are positively correlated with the local oxygen concentration, and tumor proliferation results in severe hypoxia within the tumor microenvironment (TME), leading to the development of hypoxic drug resistance in tumors.^[^ ^7^, ^8^ ^]^ In addition, the efficacy of treating deep tumors is affected more by limited drug penetration and hypoxic conditions. In multimode synergistic therapy, PTT is an oxygen‐independent cancer treatment combined with PDT to augment antitumor efficacy. Complete eradication of tumor cells requires high temperatures (>50 °C) for effective ablation; this scenario can lead to cellular necrosis and trigger the release of pro‐inflammatory factors, such as cytokine Interleukin‐6 (IL‐6), within cancerous tissues, potentially exacerbating cancer metastasis and posing risks to normal tissue health.^[^ ^9^ ^]^ However, the high heat resistance of tumor tissue under low‐temperature (<45 °C) PTT is primarily attributed to the robust self‐repair mechanisms and exceptional stress tolerance exhibited by heat shock proteins (HSPs), which diminishes the therapeutic efficacy of PTT.^[^ ^10^, ^11^ ^]^ Consequently, deep drug penetration and downregulation of HSP expression can be incorporated into drug delivery systems to augment the efficiency of multimodal therapy.^[^ ^12^, ^13^, ^14^ ^]^

As an effective cancer treatment drug, gambogic acid (GA) has made remarkable achievements in antitumor therapy.^[^ ^15^, ^16^ ^]^ Previous studies have demonstrated that GA specifically binds to the N‐terminal domain of the HSP90 protein structure,^[^ ^17^ ^]^ leading to substantial inhibition of its activation and overexpression during PTT.^[^ ^18^, ^19^ ^]^ However, the clinical application of GA is hindered by challenges such as high toxicity, poor water solubility, and limited targeting capability.^[^ ^20^ ^]^ Free small‐molecule drugs and most drug vehicles have a low delivery efficiency deep into the tumor owing to poor drug penetration and hypoxic conditions at the tumor site. Tetrahedral DNA nanostructures (TDNs) have garnered significant attention in drug delivery because of their exceptional drug‐loading capacity and customizable properties.^[^ ^21^, ^22^, ^23^ ^]^ The most important aspect is its capacity to efficiently penetrate tumor cells owing to its small size and potential.^[^ ^24^ ^]^ As carriers of GA, TDNs can significantly enhance water solubility and penetration into tumor tissue. Carbonic anhydrase (CA) IX is also overexpressed in hypoxic tumor tissues,^[^ ^25^ ^]^ thus serving as a potential tumor target. Sulphonamides have a strong affinity for CA IX, which helps the nanodelivery system provide a platform to target the deep tumor.^[^ ^26^, ^27^ ^]^

However, the transport of TDNs in blood vessels increases their susceptibility to clearance by the endothelial reticular system, and the risk of intravenous injection in vivo is a challenge in clinical practice.^[^ ^28^ ^]^ As efficient local injection delivery systems, gels exhibit the advantages of enhancing local drug concentration and facilitating rapid elimination, demonstrating significant potential in tumor therapy.^[^ ^29^, ^30^ ^]^ Low molecular weight gel (LMWG), as in a new supramolecular gel, including hydrophobic interaction, hydrogen bond, electrostatic interaction, and π‐π stacking interaction, self‐assemble into gel fibers forming 3D network structures,^[^ ^31^, ^32^ ^]^ which inhibit free diffusion to prevent the burst release of drug. ROS‐responsive gels could enhance control over drug release by incorporating ROS‐cleavable thioketal and thioether bonds.^[^ ^33^, ^34^ ^]^

**Scheme 1:**
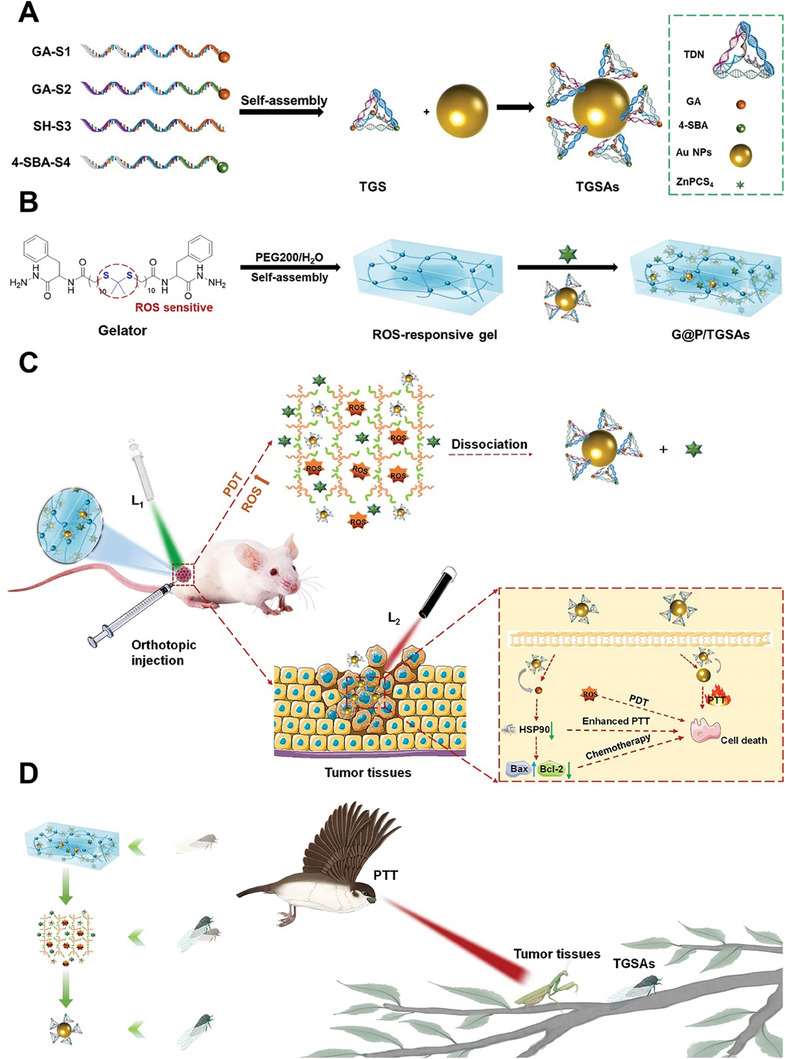
Schematic illustration of the effective synergistic treatment of tumor by chemotherapy‐PTT‐PDT based on down‐regulated HSP90 pathway. A) Synthesis of functionalized tetrahedral DNA nanostructures (TGSAs). B) Preparation process of ZnPCS_4_‐TGSAs co‐loaded gel (G@P/TGSAs). C) Mechanism of action of G@P/TGSAs in deep penetrating hypoxic tumors. D) The treatment process of G@P/TGSAs+L_1_+L_2_ was vividly shown.

Herein, we designed ROS‐responsive supramolecular gels co‐loaded with the photosensitizer Zn(II) phthalocyanine tetrasulfonic acid (ZnPCS_4_) and functionalized tetrahedral DNA (TGSAs) (G@P/TGSAs) to enhance deep tissue and cell penetration and block the HSP90 pathway to facilitate trimodal synergistic therapy (**Scheme** **1**B). The TGSAs were tetrahedral DNA modified with GA, 4‐sulfamoyl benzoic acid (SBA), and gold nanoparticles (Au NPs) (Scheme 1A). G@P/TGSAs were exposed to a 660 nm near‐infrared laser (0.1 W cm^−2^, L_1_) to generate ROS, which accelerated the degradation of the LMWG, releasing TGSAs. After the TGSAs were internalized, the tumor was irradiated with an 808 nm near‐infrared laser (0.96 W cm^−2^, L_2_). Au NPs played a role in low‐temperature PTT (<45 °C),^[^ ^35^ ^]^ where GA inhibited the expression of HSP90 in tumors to maximize the therapeutic performance of PTT (Scheme 1C). The treatment process can be aptly summarized by the phrase “Mantis catching cicada, without realizing the siskin behind.” TGSAs (cicada) escaped from the LMWG (shell) were “eaten” by tumor tissues (mantis), and the tumor tissues were “killed” by PTT therapy (siskin) (Scheme 1D, authorized by Figdraw). The “Cicada Out of the Shell” drug delivery system was expected for enhanced chemotherapy‐PDT‐PTT synergistic therapy.

## 2 Results and Discussion

### 2.1 Synthesis and Characterization of TGSAs

**Figure 1:**
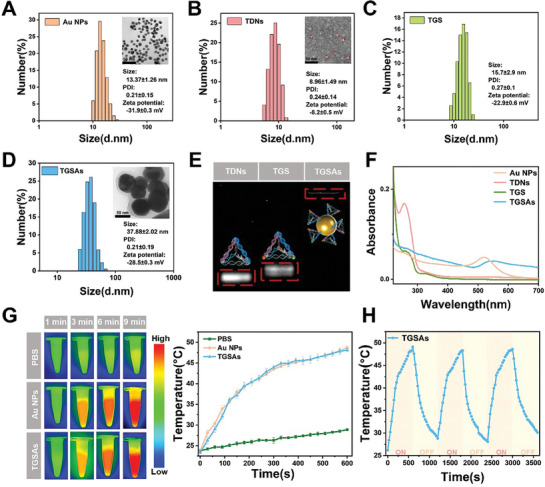
Size distribution, zeta potential, and TEM images of A) Au NPs, B) TDNs, C) TGS, and D) TGSAs. E) Agar‐gel electrophoresis images of purified TDNs, TGS, and TGSAs. F) UV‐vis‐NIR absorption spectrum of Au NPs, TDNs, TGS, and TGSAs. G) NIR thermal imaging photos of PBS, Au NPs, and TGSAs and plotted photothermal curves. H) Photothermal stability curves of TGSAs within 3600 s.

In this study, functionalized tetrahedral DNA nanostructured TGSAs were developed to target hypoxic tumors. The functionalized oligonucleotide chains were purified by ultrafiltration, and electrospray ionization mass spectrometry (ESI‐MS) and agarose gel electrophoresis verified the synthesis. The successful assembly of TGSAs was investigated using agarose gel electrophoresis, dynamic light scattering (DLS), ultraviolet‐visible near‐infrared absorption spectrum (UV‐vis‐NIR absorption spectrum), and transmission electron microscopy (TEM). Agarose gel electrophoresis revealed that the molecular weight of the modified functional DNA oligonucleotide was greater than that of the corresponding unmodified DNA single strand (Figure S1, Supporting Information). The relatively backward position of the TGSAs in the imaging results indicated their successful synthesis (**Figure** **1**E), and the purity of the modified functional DNA oligonucleotides and TGSAs was more than 90%. As shown in Figure 1A–D, Au NPs, TDNs, TDNs‐GA‐SBA (TGS), and TGSAs all showed good particle size and distribution, and the size of the TGSAs was 37.88 ± 2.2 nm with the zeta potential was −28.5 ± 0.3 mV. The UV‐vis‐NIR absorption spectra (Figure 1F) further show that the Au NPs had an absorption peak at ≈520 nm, and the TDNs had an absorption peak at ≈255 nm. The absorption peaks of TGS are ≈260 and 325 nm, which contain the characteristic absorption peaks of TDNs and redshift, respectively, proving the successful synthesis of TGS. TGSAs have maximum absorptions at 263 and 550 nm, consistent with the characteristic absorption peaks of TGS and Au NPs, respectively. The absorption peak of TGSAs produces a redshift compared to the previous peak position, which proves the successful synthesis of TGSAs. Figure 1G showed that compared to phosphate buffered saline (PBS), Au NPs and TGSAs exposed to L_2_ for 9 min reached 47.2 and 47.3 °C, respectively, indicating that TGSAs exhibited excellent photothermal conversion efficiency after laser irradiation. In addition, the TGSAs were exposed to L_2_ and cooled. After three repetitions, the TGSAs still had excellent photothermal efficiency, indicating that TGSAs had excellent photothermal stability (Figure 1H).

### 2.2 Synthesis and Characterization of Gelator

**Figure 2:**
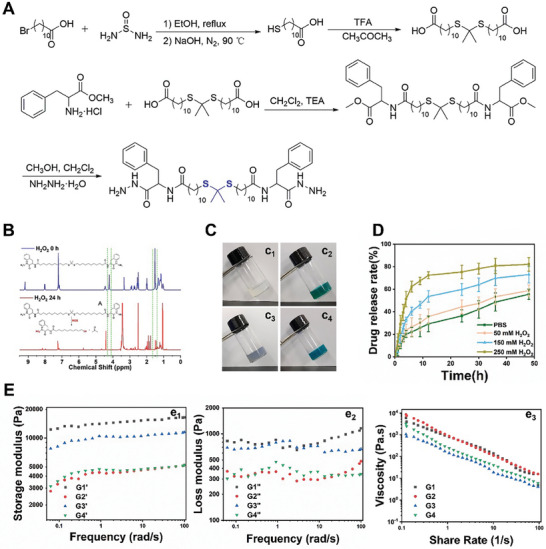
A) Synthesis route of the gelator. B) ROS responsiveness of the gelator was verified by ^1^H NMR. C) Pictures of c_1_) G1, c_2_) G2, c_3_) G3, and c_4_) G4. D) Drug‐release curves of G@Cy5‐TGSAs at different concentrations of H_2_O_2_. E) Rheological properties of gels: e_1_) Energy storage modulus (G′), e_2_) loss modulus (G″), and e_3_) viscosity‐shear rate curves.

**Figure** **2**A shows the synthetic route of the ROS‐responsive gelator, which was successfully synthesized in a previous study.^[^ ^36^ ^]^ The structures of thioketal (TK), gelator precursor (Phe‐TK‐Phe), and the gelator were characterized by proton nuclear magnetic resonance (^1^H NMR) spectroscopy. The position of the characteristic TK peak (‐C(CH_3_)_2_‐) was 1.582 ppm, which proved that the thioketone was successfully prepared (Figure S2, Supporting Information). The characteristic peak of methyl proton (PhCH_2_CH‐) was 4.903 ppm, demonstrating the successful preparation of the gelator precursor Phe‐TK‐Phe (Figure S3, Supporting Information). The CH_3_O peak was 3.730 ppm but disappeared in the gelator spectrum. In addition, the positions of the other peaks in the spectrum were consistent with those of the gelator structure, indicating that the ROS‐responsive gelator was successfully synthesized (Figure S4, Supporting Information).

Moreover, the ROS responsiveness of the gelator was characterized using ^1^H NMR spectroscopy. Compared to the gelator, the methyl proton peak (1.51 ppm) and amino proton peak (4.22 ppm) disappeared after co‐incubation with the Fenton reagent for 24 h (Figure 2B), which proved that the gelator had ROS responsiveness.

### 2.3 Preparation of Blank and Drug‐Loaded Gel

The gelator exhibits excellent glue‐forming properties in solvents such as ethanol, ethylene glycol, polyethylene glycol (PEG)200, and PEG400. The gelator was heated, stirred, and cooled to room temperature (RT) in PEG200 and H_2_O (3:2) to form the gel. The 3D network structure inside the gel in ethanol (10 and 15 mg mL^−1^) and chloroform (10 and 15 mg mL^−1^) is shown in the scanning electron microscope (SEM) images (Figure S7, Supporting Information). The critical gel concentration (CGC) was determined by testing the minimum dose of gelator required when the gel was cooled to 37 °C (Table S2, Supporting information). To evaluate the gel formation state and stability, rheological tests were performed on the following gels and drug‐loaded gels: Blank gel (G1), ZnPCS_4_‐loaded gel (G2), TGSAs‐loaded gel (G3), and ZnPCS_4_‐TGSAs co‐loaded gel (G4) (Figure 2C–c_1–_c_4_). There is a charge interaction between the sulfonic acid group in the ZnPCS_4_ structure and the amino group in the gelator structure, and there is a benzene ring structure in the TGSAs structure, which forms a strong π–π stacking force with the gelator. These strong non‐covalent forces enable the stability of drug molecules in the 3D network of the gel. The storage moduli (G’) of the four gels were higher than the loss moduli (G’), which verified the existence of the gel form and its excellent stability (Figure 2E–e_1_,e_2_). G4 had a lower storage modulus, which may be because the gel network became more disordered because of the addition of the two contents, which led to the weakening of the rheological properties of the gel. The viscosity of all gels decreased with an increase in the shear rate, which showed that the gels exhibited shear thinning behavior, ensuring the characteristics of in situ injection at the tumor. The forms of G1 and G4 in vivo are shown in Figure S11, Supporting Information.

### 2.4 Study on Gelation Mechanism and Molecular Dynamics Simulation of Gelator

^1^H NMR and fourier transform infrared (FT‐IR) spectroscopy were used to verify the intermolecular forces of the gelator and the mechanism of gel formation. With increasing gel concentration, the chemical shift of the amide bond moved to a lower field (from 7.919 to 7.931 and from 6.514 to 6.520 ppm), and the chemical shift of the benzene ring shifted to a higher field (from 7.279 to 7.277 ppm) (Figure S5, Supporting Information). The hydrogen bond and π–π stacking force that existed in the molecular structure of the gelator were demonstrated in the results of the spectrogram. They were important driving forces for transforming the gelator into a gel. FT‐IR and local magnification images of the gelator and xerogel at different concentrations are shown in Figure S6, Supporting Information to verify the intermolecular forces further. Compared with the FT‐IR spectra of the gelator, the tensile vibration peak of the amino N–H bond of the xerogel with a concentration of 10 mg mL^−1^ was transferred from 3296.5 to 3293.8 cm^−1^, and the vibration peak of the xerogel with a concentration of 25 mg mL^−1^ was transferred from 3296.5 to 3291.1 cm^−1^. At the same time, the vibration peak of the xerogel with a concentration of 25 mg mL^−1^ was transferred from 1641.6 to 1640.8 cm^−1^, and the frequency of the N–H deformation vibration peak of 10 mg mL^−1^ xerogel was 1536.6 cm^−1^, and the frequency of the vibration peak of 10 mg mL^−1^ dry gel was 1537.2 cm^−1^. The existence of hydrogen bonding forces between the molecules was further verified. The change in the methylene vibration peak indicated that the alkyl chains in the gelator were stacked, and van der Waals forces existed. Therefore, the hydrogen bond force and van der Waals force become the vital forces driving the self‐assembly of the gel, which is constituted of hydrogen bond force and van der Waals force.

**Figure 3:**
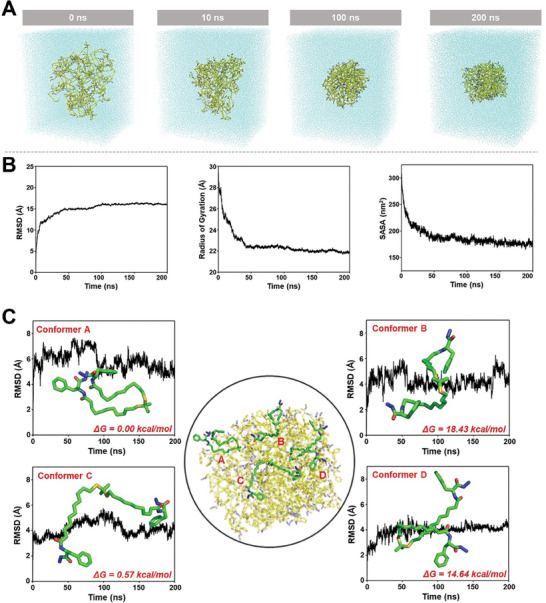
Molecular dynamics simulation of gelator structure. A) Snapshot of the system formed by gelator within 200 ns B) RMSD, gyratory radius, and SASA values. C) Free energy calculation value of gelator structure different isomers (Conformer A, Conformer B, Conformer C, Conformer D).

To investigate the stability of the gelator structure and the driving force for gel formation, molecular dynamics (MD) simulations were performed. An NPT‐integrated system was used to simulate the self‐assembly of the gelators. As shown in **Figure** **3**A, at 0 ns, the initial state of the gelator was randomly distributed throughout the system. The root‐mean‐square deviation (RMSD), Radius of Gyration (Rg), and solvent‐accessible surface area (SASA) of the gelator gradually stabilized over time (Figure 3B). These results indicated that the gelator structure gradually stabilized. Finally, the free energies of different gelator isomers were calculated (Figure 3C). The calculation results exhibited that conformer A has the lowest free energy among the four isomers at 37 °C, so it can be considered the most energy‐stable gelator structure configuration. This configuration of the gelator structure had excellent stability and could drive the gels to construct a 3D network structure.

### 2.5 Drug Release Properties In Vitro

As shown in Figure 2D, the responsive cleavage‐release behavior of Gel@Cy5‐TDNs‐GA‐SBA‐Au NPs (G@Cy5‐TGSAs) was observed in the presence of H_2_O_2_. With increasing H_2_O_2_ concentration, the drug release rate gradually accelerated. After 6 h, the cumulative release rate of G@Cy5‐TGSAs in the medium containing 250 mM H_2_O_2_ exceeded 65%, whereas that in the medium without H_2_O_2_ was only 22%. The drug release rate of G@Cy5‐TGSAs under H_2_O_2_ treatment reached more than 80% after 48 h, which fully demonstrated its ROS‐responsive behavior.

### 2.6 Cytotoxicity Assay

**Figure 4:**
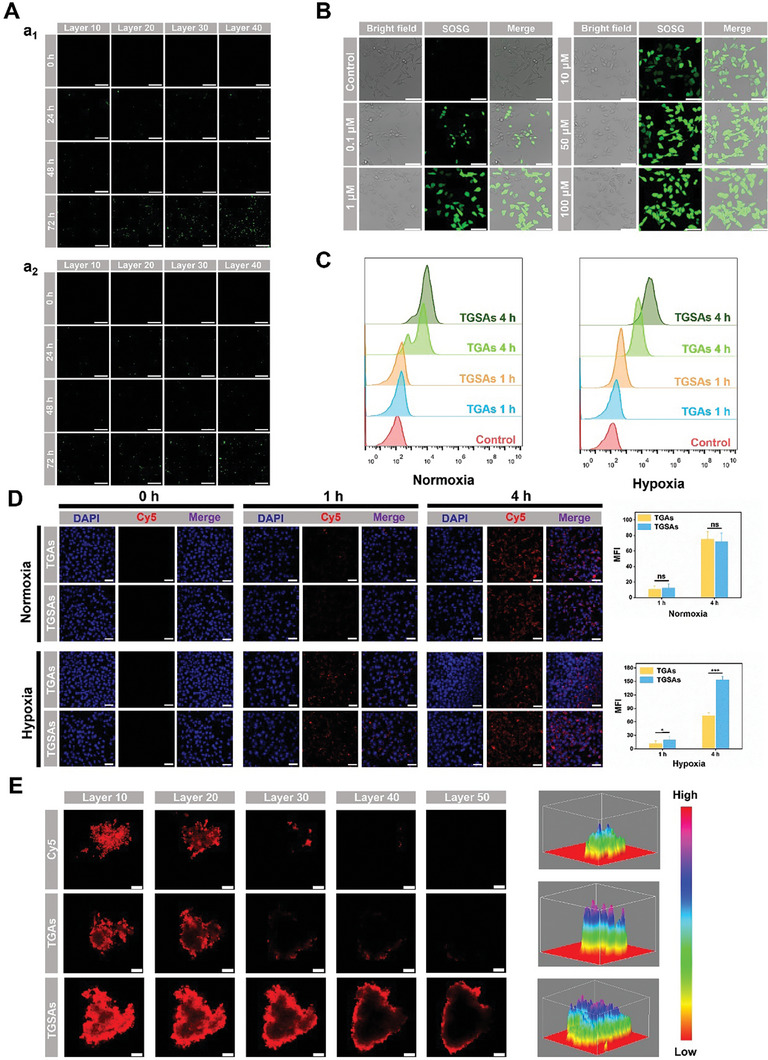
A) CLSM imaging verified the survival of a_1_) 4T1 cells and a_2_) L929 cells inside the gel. The scale of each drawing was 25 µm. B) CLSM verified ROS production at different concentrations of ZnPCS_4_ in vitro. The scale of each drawing was 25 µm. C) FCM and D) CLSM were used to analyze the uptake of different nanostructures quantitatively and qualitatively by 4T1 cells under normal and anaerobic conditions, respectively. Semi‐quantitative analysis of CLSM imaging results directly demonstrated the statistical differences in the uptake of the two types of cells under different states. The scale of each drawing was 50 µm. E) CLSM imaging and 3D analysis images results after the materials were incubated with multicellular spherules. The scale of each drawing was 75 µm. Mean ± SD, *n* = 3 (*p < 0.05, ***p < 0.001).

The cytotoxicity of blank TDNs, gelator and gel against L929 and 4T1 cells was evaluated using the MTT and live/dead assays, respectively. The effects of different laser types (L_1_ and L_2_) and laser duration on cell survival were evaluated. The type and duration of laser exposure were not toxic to either cell line (Figure S8, Supporting Information). The good biocompatibility of the TDNs was proven by the fact that the cell viability could still reach more than 80% at TDNs concentrations up to 500 nM (Figure S9, Supporting Information). In addition, MTT assay results showed that the blank gel extract had good biocompatibility (Figure S10, Supporting Information). Confocal laser scanning microscope (CLSM) scans at different levels showed that 4T1 cells (**Figure** **4**A–a_1_) and L929 cells (Figure 4A–a_2_) grew well in the gel and proliferated and migrated in the 3D network, which may be related to the nutritional properties of L‐phenylalanine methyl ester. These results indicated that the gel can be used as a safe and effective carrier for drug delivery.

### 2.7 ROS Level Assay In Vitro

When the photosensitizer ZnPCS_4_ was irradiated with L_1_, it produced a large amount of ROS,^[^ ^37^ ^]^ promoting the cleavage of the gel and cell death. Different concentrations of ZnPCS_4_ were exposed to L_1_, and the production of ROS (green fluorescence) was verified using CLSM. The results showed that ZnPCS_4_ significantly induced ROS production in a concentration‐dependent manner (Figure 4B).

### 2.8 Evaluation of Drug Penetration In Vitro

The permeability of TGSAs in vitro was evaluated by constructing multicellular tumor spheres (MTSs). The CLSM results showed that the fluorescence intensity of MTSs incubated with TGSAs was stronger than that of MTSs incubated with free Cy5 and TDNs‐GA‐Au NPs (TGAs), which was more intuitively observed in the topological 3D view images (Figure 4E). This may be attributed to the stronger tissue permeability of the DNA nanostructures and the active targeting of TGSAs.

### 2.9 Evaluation of Cellular Uptake and Endolysosomal Escape

**Figure 5:**
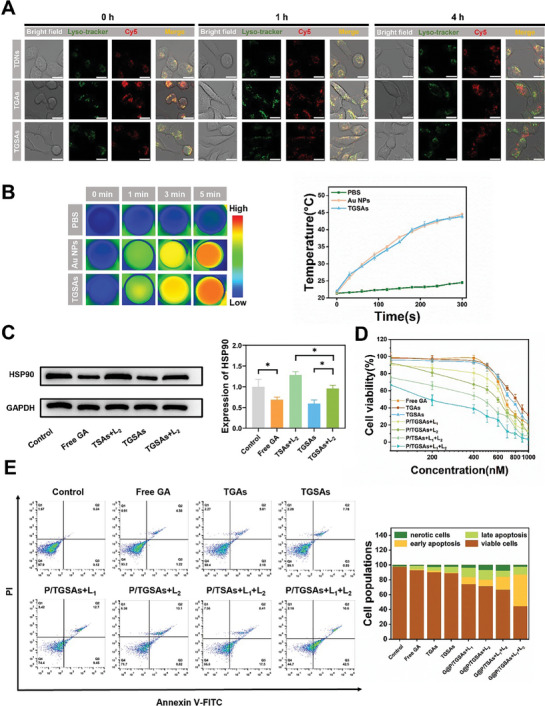
A) Lysosome escape and semi‐quantitative analysis of Cy5‐modified TDNs, TGAs, and TGSAs at 0, 1, and 4 h, respectively. The scale of each drawing was 25 µm. B) In vitro photothermal near‐infrared thermal imaging photographs and plotted photothermal curves of PBS, Au NPs, and TGSAs. C) The effects of different groups on HSP90 expression were verified by Western blot assay. D) Cell viability and E) apoptosis of 4T1 cells after treatment in each experimental group were shown. Mean ± SD, *n* = 3 (*p < 0.05, **p < 0.01).

Sulfonamides can form coordination bonds with metal ions in the active center of CA IX overexpressed by hypoxic tumors, and the stable conformation helps establish an excellent active targeting platform in TDNs.^[^ ^38^ ^]^ The CLSM results indicated a time‐dependent uptake of red fluorescence (Cy5‐modified TGAs or TGSAs) (Figure 4D). Notably, cells under hypoxic conditions internalized more TGSAs than those under normal oxygen conditions, attributed to the targeting of SBA to the hypoxic tumor cells. The flow cytometry (FCM) results are consistent with the CLSM results (Figure 4C). It has been reported that TDNs are transferred to lysosomes after cellular uptake, which greatly reduces drug availability; however, after targeted modification, TDNs can escape from lysosomes.^[^ ^39^ ^]^ At 0 and 1 h, most of the nanostructures were still located in the lysosomes, with yellow fluorescence (the overlap of red fluorescence (Cy5‐modified TGAs or TGSAs). Green fluorescence (LysoTracker). With the extension of incubation time, the red and green fluorescence were separated, proving that TGAs and TGSAs successfully escaped from the lysosome (**Figure** **5**A).

### 2.10 Photothermal Effect and HSP90 Expression Detection In Vitro

Photothermal images and curves of 4T1 cells incubated with different materials were recorded using a thermocouple thermometer and near‐infrared thermal imager. Compared to PBS, cells incubated with Au NPs and TGSAs exhibited excellent photothermal properties. The photothermal curve showed that the temperature of TGSAs incubated cells could reach 44.5 °C after L_2_ irradiation for 5 min, which was the appropriate temperature for PTT (Figure 5B). Mild‐temperature PTT minimized the leakage of intracellular inflammatory factors and prevented severe malignant reactions when HSP90 was overexpressed. GA could down‐regulate the overexpression of HSP90 and help to enhance the therapeutic effect of PTT. As shown in Figure 5C, compared to the control group, the intracellular expression of HSP90 was significantly inhibited by free GA. HSP90 was overexpressed in the TDNs‐SBA‐Au NPs (TSAs)+L_2_ group compared to that in the TGSAs+L_2_ group, whereas HSP90 was downregulated in the TGSA group. Semi‐quantitative analysis showed that TGSAs could downregulate the overexpression of HSP90 to improve the sensitivity of tumor cells to PTT and could become an effective treatment method for tumors.

### 2.11 Antitumor Activity In Vitro

The 4T1 cells were incubated differently to evaluate the antitumor efficiency of different materials and lasers. The results (Figure 5D) showed that the IC50s of all groups (Free GA, TGAs, TGSAs, P/TGSAs (ZnPCS_4_/TGSAs) +L_1_, P/TGSAs+L_2_, P/TSAs (ZnPCS_4_/TSAs) +L_1_+L_2_, and P/TGSAs+L_1_+L_2_) were 0.675, 0.816, 0.745, 0.651, 0.601, 0.466, and 0.188 µM. The results indicated that the antitumor efficiency of TGSAs was significantly improved after exposure to L_1_ and L_2_ respectively.

### 2.12 Cell Apoptosis

The apoptosis rates of 4T1 cells treated with different formulations were investigated by staining with FITC‐Annexin V and propidium iodide. As shown in Figure 5E, the apoptosis rates of P/TGSAs+L_1_ and P/TGSAs+L_2_ were significantly higher than those of Free GA, and the percentage of apoptosis increased to 22.15% and 21.92%, which were 3.88 and 3.84‐fold higher than those in the free GA group, respectively. Excitingly, the P/TGSAs+L_1_+L_2_ group significantly increased the apoptosis rate of the cells, which was about 55.28%. The treatment effect of P/TGSAs+L_1_+L_2_ was superior to that of any single therapy, proving that the combination of trimodal synergistic therapies had an efficient anticancer ability.

### 2.13 Proteomic Analysis of the Regulation‐Related Proteins in Tumor Cells

**Figure 6:**
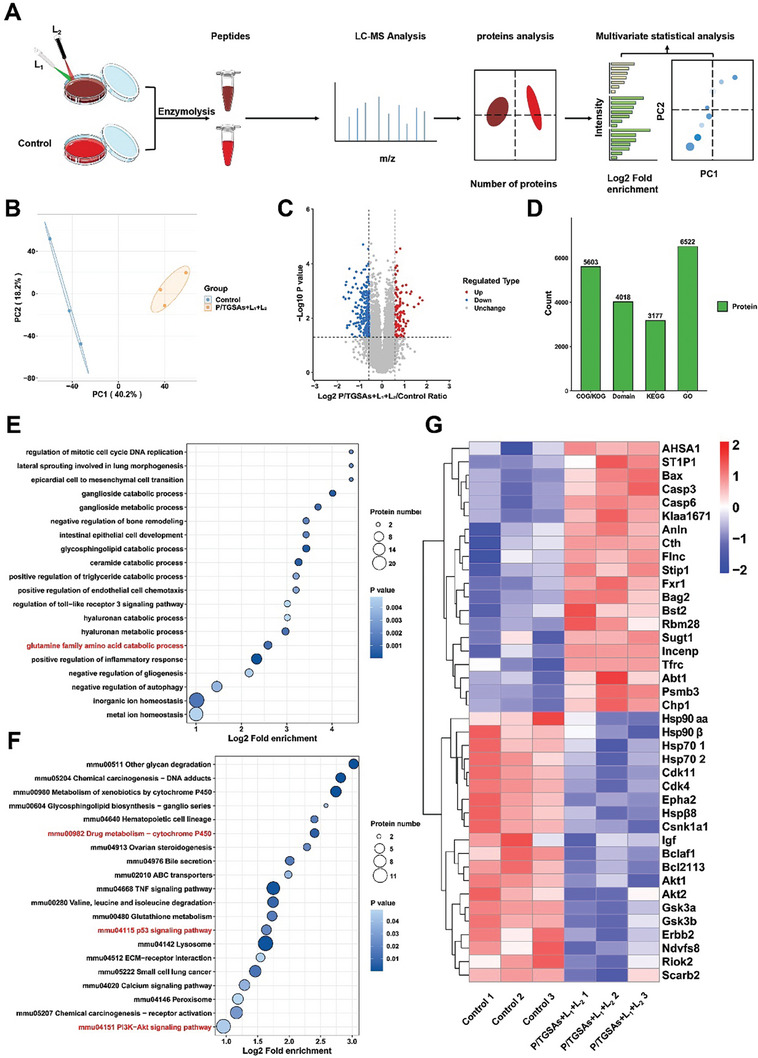
Downregulation of HSP90 and apoptosis induced by P/TGSAs+L_1_+L_2_ in 4T1 cells. A) Diagrammatic sketch of proteomic analysis. B) Visual principal component Analysis (PCA) images based on the relative quantitative values of all samples. C) Volcano map of differences in protein expression caused by P/TGSAs+L_1_+L_2_ compared with the control group. D) Gene Ontology (GO), protein domain, KEGG pathway, and COG/KOG function provide comprehensive functional annotation of the identified proteins. The differences in the expressed proteins induced by the P/TGSAs+L_1_+L_2_‐treated group compared to the control group were analyzed by E) Biological Process of Gene Ontology (GO) and F) KEGG pathway. G) Expression of differential proteins in P/TGSAs+L_1_+L_2_‐treated and control group in a heat map.

Proteomic analysis was used to detect changes in proteins and pathways in the tumor cells after P/TGSA +L_1_+L_2_ treatment (**Figure** **6**A). First, changes in intracellular protein levels after treatment were evaluated, and the principal component analysis (PCA) results (Figure 6B) showed a significant difference in protein expression between the P/TGSA +L_1_+L_2_ treatment group and the control group. The volcano map showed that P/TGSA +L_1_+L_2_ treated cells had more differentially expressed proteins than the control group (Figure 6C), where the red dots represent significant upturns, blue dots represent significant downturns, and gray dots indicate no significant differences. Gene Ontology (GO), protein domain, Kyoto Encyclopedia of Genes and Genomes (KEGG) pathway, and Clusters of Orthologous Groups of proteins/euKaryotic Ortholog Groups (COG/KOG) functional classifications were used to annotate the identified proteins to understand their functional properties (Figure 6D). Figure 6E,F show the enrichment analysis of differential protein function using the Biological Process of GO and KEGG pathways, respectively. Inhibition of HSP90 may affect the metabolism of glutamine family amino acids and the cytochrome P450, P53, and PI3K signaling pathways, which may affect the process of apoptosis. Differences in the expression of multiple proteins between the two groups were further visualized using a heat map (Figure 6G). HSP90‐associated protein kinases, such as Cdk4, Akt1, Cdk4, and Csnk1a1, were downregulated, which may cause protein misfolding or degradation. Additionally, the expression levels of apoptosis‐related proteins (Bax, caspase3, caspase6) expressed in the P/TGSA +L_1_+L_2_ treated group were significantly higher than those in the control group. These results suggested that P/TGSAs+L_1_+L_2_ treated cells may downregulate heat shock response‐related proteins and genes, reducing heat stress in tumor cells. In addition, the apoptotic pathway was activated, confirming the synergistic trimodal therapeutic effect of P/TGSA exposed to lasers.

### 2.14 Evaluation of Antitumor Effect In Vivo

**Figure 7:**
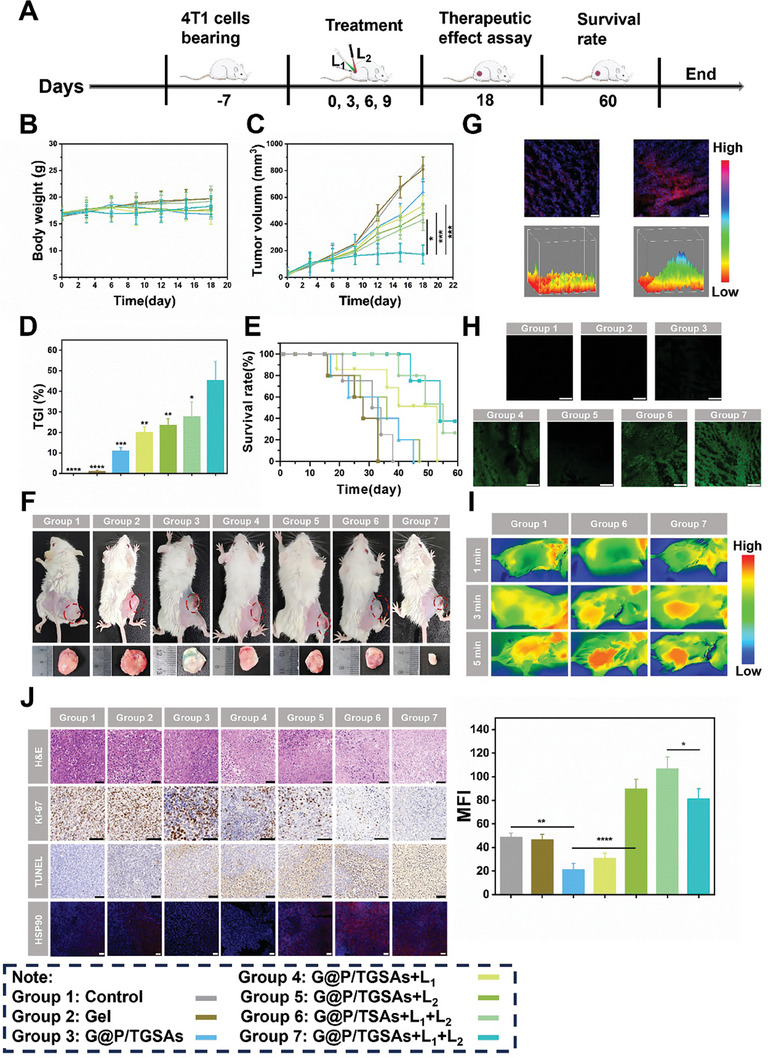
Antitumor effect of each experimental group on tumor‐bearing mice. A) Flowsheet of the treatment regimen for tumor‐bearing mice. B) Body weight, C) tumor volume, D) tumor growth inhibition value (TGI), and E) survival curve of mice in each experimental group. F) Tumor‐bearing mice and tumor photos of each experimental group were randomly selected. G) Permeation of free Cy5 and G@P/ Cy5‐TGSAs in vivo, on a scale of 50 µm. H) ROS production in vivo after treatment in different groups, the scale of each drawing was 25 µm. I) Thermal imaging photos of different groups of treated mice at 1, 3, and 5 min. J) H&E, Ki‐67, TUNEL histochemistry results, and immunofluorescence of HSP90 in vivo and its semi‐quantitative analysis, the scale of each drawing was 50 µm.

The 4T1 tumor‐bearing mice were randomly divided into seven groups: Control, gel, G@P/TGSAs, G@P/TGSAs+L_1_, G@P/TGSAs+L_2_, G@P/TSAs+L_1_+L_2_, and G@P/TGSAs+L_1_+L_2_. A flowchart for evaluating the anti‐tumor effect in vivo is shown in **Figure** **7**A. The weights of the mice in each group increased to different degrees (Figure 7B), indicating that the drug delivery system had good biocompatibility. As shown in Figure 7C, a significant inhibition of tumor growth in the G@P/TGSA+L_1_+L_2_ group was observed. The tumor volume in the G@P/TGSAs+L_1_+L_2_ group was the smallest after treatment, and the tumor growth inhibition (TGI) value was as high as 45% (Figure 7D,F). The survival rate of the mice in the G@P/TGSA+L_1_+L_2_ group was as high as 40% on day 60, which was much higher than that in the other groups (Figure 7E).

The permeability of the TGSAs was also examined in vivo. The CLSM imaging results (Figure 7G) showed that only a small amount of fluorescence was present in the tumor slices of mice injected with Cy5. However, a large area of red fluorescence was observed in the tumor slices after the mice were injected with TGSAs. The difference in the fluorescence intensity between the two groups was visually demonstrated in a topological 3D view. These results showed that TGSAs can penetrate the extracellular matrix and penetrate deep into the tumor tissue, whereas free drugs can only penetrate the superficial surface of the tumor tissue.

The singlet oxygen sensor green (SOSG) reagent was used to detect ROS production and evaluate the effect of PDT in vivo. Compared to the control group, the G@P/TGSAs+L_1_, G@P/TSAs+L_1_+L_2_, and G@P/TGSAs+L_1_+L_2_ groups exhibited strong green fluorescence (Figure 7H). The results showed that the drug delivery system containing ZnPCS_4_ exhibited excellent ROS generation efficiency after exposure to L_1_.

PTT efficiency of G@P/TGSAs+L_1_+L_2_ in vivo was evaluated using a near‐infrared thermal imager after in situ injection (Figure 7I). After the tumors were irradiated with L_2_ for 5 min, the temperature of the tumor site in the control group changed little. In contrast, the temperature of the G@P/TSAs+L_1_+L_2_ and G@P/TGSAs+L_1_+L_2_ groups increased rapidly to 42.7 °C and 44.1 °C, which proved that the nanostructures had excellent PTT efficiency in vivo.

**Figure 8:**
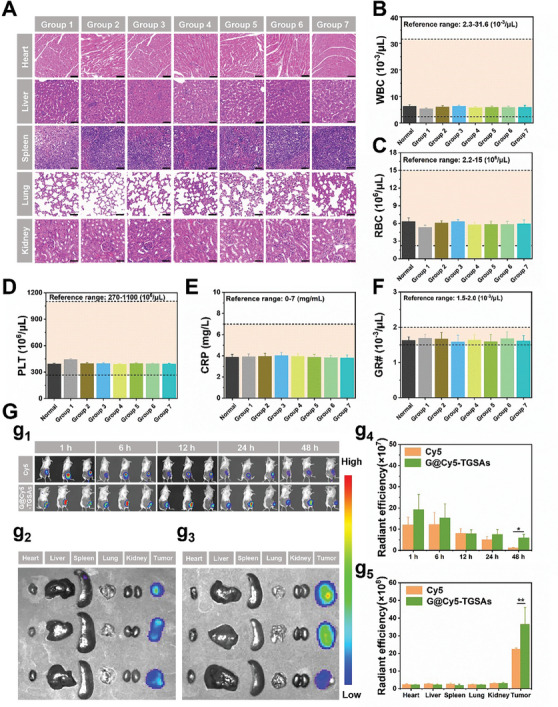
A) Immunohistochemical section results of the heart, liver, spleen, lung, and kidney of mice in each experimental group, the scale of each drawing was 50 µm. Blood biochemical indicators included B) white blood cell count (WBC), C) red blood cell count (RBC), D) blood platelet count (PLT), E) C‐reactive protein (CRP), and F) neutrophil granulocyte (GR#). G) In vivo elimination in mice with time after in situ injection. g_1_) Fluorescence intensity distribution and elimination in vivo. Fluorescence intensity in isolated organs and tumors injected with g_2_) Cy5 and g_3_) G@Cy5‐TGSAs. g_4_) Fluorescence intensity analysis in vivo and g_5_) isolated organs. Mean ± SD, *n* = 3 (*p < 0.05, **p < 0.01).

The antitumor efficacy of G@P/TGSAs+L_1_+L_2_ was further studied by hematoxylin‐eosin (H&E) staining of the tumor slices (Figure 7J). The results showed that the G@P/TGSAs+L_1_+L_2_ group exhibited the most severe nuclear deformation and fragmentation. In addition, immunohistochemical analysis revealed a few Ki‐67‐positive proliferating cells and many TUNEL‐positive apoptotic cells were observed in the tumor tissues of the G@P/TGSAs+L_1_+L_2_ group, which proved that G@P/TGSAs+L_1_+L_2_ had the strongest anti‐tumor effect. The immunofluorescence results (Figure 7J) showed that G@P/TGSAs+L_1_+L_2_ effectively down‐regulate the expression of HSP90 in vivo. H&E staining of the original organs and blood analysis of the mice after treatment are important indicators for evaluating the safety of drug delivery systems. The results (**Figure** **8**B–F, and Table S3, Supporting Information) showed that all biochemical indicators were within the normal range, proving that the developed drug delivery systems were safe and effective.

### 2.15 Biodistribution and Degradation Characteristics

In vivo imaging was used to verify the distribution and degradation of the nanostructures in vivo. Free Cy5 and G@Cy5‐TGSAs were injected into the tumor sites of mice, and the fluorescence intensity signals were detected at different times (Figure 8G–g_1_). The results showed that both materials were distributed at the tumor sites. At 48 h, the fluorescence intensity of G@Cy5‐TGSAs was stronger than that of free Cy5, which could be attributed to the slow‐release properties of the gel. Free Cy5 was rapidly metabolized by the body, as verified by organ imaging ex vivo (Figure 8G–g_2_,g_3_). The results of fluorescence intensity analysis (Figure 8G–g_4_,g_5_) were consistent with the above results. In summary, the G@Cy5‐TGSAs gel system plays a therapeutic role at the tumor site and ensures local drug concentration and continuous release.

## 3 Conclusion

In this study, we designed ROS‐responsive supramolecular gels co‐loaded with the photosensitizer Zn(II) phthalocyanine tetrasulfonic acid (ZnPCS_4_) and functionalized tetrahedral DNA (TGSAs) (G@P/TGSAs) for enhanced deep tissue and cell penetration, and blockage of the HSP90 pathway to facilitate trimodal synergistic therapy. The results exhibited intermolecular forces, including hydrogen bond, π‐π stacking, and van der Waals force, which were the driving forces of gel formation. After L_1_ irradiation, the gel exhibited ROS‐stimulation response properties and could be used for the controlled release of ZnPCS_4_ and TGSAs. In addition, the 4‐SBA‐mediated targeting and strong permeability of TDNs could enhance the deep targeting and permeability of TGSAs to tumors. After L_2_ irradiation, the TGSAs exerted PTT on the tumor and inhibited the overexpression of HSP90, thus overcoming the heat resistance of the tumor. In summary, the G@P/TGSAs system achieved the spatiotemporal release of nanostructures at the tumor site and had a synergistic effect of trimodal synergistic therapy. This study provides a new approach for applying gel and tetrahedral DNA nanostructures in clinical settings.

## 4 Experimental Section

### Materials

The single strands of DNA nucleotides with amino and sulfhydryl groups respectively used in this experiment were all from Shanghai Sheng Gong Biotechnology (China) Co., LTD. (Table S1, Supporting Information). Mouse fibroblast L929 and mouse breast cancer 4T1 cells were purchased from the Cell Bank of the Chinese Academy of Sciences (Shanghai, China). 11‐Bromoundecanoic acid, L‐phenylalanine methyl ester hydrochloride, H_2_O_2_, PEG200, GA, and 6‐bromocaproic acid were acquired from Shanghai Aladdin Biochemical Technology Co., Ltd. (China). Hydrazine hydrate was purchased from Sinopod Chemical Reagent Co., Ltd. N, N’‐carbonyldiimidazole (CDI), 2‐(7‐azobenzotriazole)‐N, N, N', N' ‐tetramethylurea hexafluorophosphate (HATU), N, N‐diisopropylethylamine (DIPEA), N, N‐dimethylacetamide (DMA), boric acid buffer (pH 9.0), RNase free sodium chloride solution (5 M), gold (III) chloride trihydrate, sodium citrate dihydrate, and 4‐sulfamylbenzoic acid (SBA) were purchased from Shanghai McLean Biochemical Technology Co., Ltd. (China). SOSG reagent was obtained from Dalian Meilun Bioscience Co., Ltd. (Dalian, China). Lyso‐Tracker Green, 2',7'‐Dichlorodihydrofluorescein diacetate (DCFH‐DA), and 2‐(4‐amidinophenyl)‐6‐indolecarbamidine dihydrochloride (DAPI) were purchased from Shanghai Biyuntian Biotechnology Co., Ltd. (China), ZnPCS_4_ come from Shanghai Haohong Biomedical Technology Co., Ltd. (China). All reagents could be used without further purification.

### Characterizations

The successful synthesis of the structures of all products and the ROS‐responsive properties of the gelator were verified by ^1^H NMR (JNM ECZ600R/S1, Japan). The mechanism of gelation was verified by ^1^H NMR and FT‐IR (Nicolet 6700, USA). The SEM (Sigma500, Germany) was also used to characterize the microstructure of the gel. The rheological properties of gels were tested by Advanced Rheology Expanded Systems (Malvern Kinexus lab+ system, UK). The release properties of gels were measured by fluorescence spectroscopy (F‐7100, Japan). The morphology of nanostructures was observed by TEM (HT7700, Japan). The UV spectrum of nanostructures was detected using a DU640 UV spectrophotometer (Beckman Coulter, USA). The hydromechanical diameter and zeta potential of nanostructures were determined using DLS (Nano ZS90, UK). Cytotoxicity was detected using a microplate detector (BioTek, USA). Immunofluorescence, cellular uptake, and lysosome escape behavior were observed by CLSM (Leica, Germany).The Western blotting and agarose gel electrophoresis were performed with the Chemi Doc XRS+ system (Bio‐Rad, USA). Cellular uptake behavior and apoptosis were characterized by a FCM (CytoFLEX S, China). IVIS Spectrum (PerkinElmer, USA) was observed for in vivo imaging. The results of immunohistochemistry were observed by a Panoramic tissue cell scanning analyzer (Pannoramic MIDI, Hungary).

### Synthesis of ROS‐Responsive Gelator

The general procedure for synthesizing ROS‐responsive gelators is shown in Figure 2A. The detailed processes are presented in the Supporting Information. In brief, phenylalanine methyl ester fragments were attached to both ends of a synthesized ROS‐responsive molecule (TK) to form a Phe‐TK‐Phe. Finally, hydrazine hydrate reacted with Phe‐TK‐Phe to obtain the ROS‐responsive gelator (molecular weight: 799).

### Synthesis of TGSAs—Synthesis of AuNPs

A HAuCl_4_ solution (10 mg mL^−1^, 1.4 mL) was dissolved in boiled water (28.6 mL). A sodium citrate solution (11.5 mg mL^−1^, 3 mL) was quickly added to the mixture. Then, the solution was sustained for 30 min and stored at 4 °C. The Au NPs were characterized using UV–vis‐NIR spectroscopy, TEM and DLS.

### Synthesis of TGSAs—Synthesis of Functionalized DNA Oligonucleotides

GA (1.57 mg, dissolved in 12.5 µL DMA, 2500 nmol, 25 eq) was added to the solution containing HATU (0.95 mg, 400 mM, dissolved in 6.25 µL DMA, 2500 nmol, 25 eq) and DIPEA (6.25 µL, 400 mM, dissolved in DMA, 2500 nmol, 25 eq) and performed at 0–10 °C for 20 min. The above mixture was subsequently added to NH_2_‐S1 (100 nmol, 1 equiv) dissolved in a borate buffer solution (200 µL, 500 mM, pH 9.5) for 20 min at RT. The above composites were added to the mixture of aqueous NaCl solution (25 µL, 5 M) and anhydrous ethanol (295 µL) to continue the reaction for 1 h. Sequentially, the intermediates were stored at −20 ° C for 3 h, filtered, dialyzed, and concentrated to obtain GA‐S1 (100 µM). The GA‐S2 and SBA‐S4 syntheses were similar to those described above. Functional DNA oligonucleotides were verified using ESI‐MS and agarose gel electrophoresis.

### Synthesis of TGSAs—Synthesis of TGSAs

The equimolar quantities of modified DNA oligonucleotides (GA‐S1, GA‐S2, SBA‐S4) and S3 were mixed and heated to 95 °C for 15 min. Then, the solution rapidly cooled to 4 °C overnight to gain TGS. TGS solution (1 µM, 200 µL) and Au NPs solution (1 mL) were mixed and gently stirred overnight at 26 °C. Then, NaCl solution (3 M, 100 µL) was added to the above mixture per 1 h, and the solution was placed at 4 °C overnight after 10 h. After centrifugation (13 000 rpm, 30 min) of the mixed solution, NaCl solution (100 mM, 100 µL) was used to disperse precipitation and centrifugation again. Finally, TGSAs solution (1 µM) was obtained by dispersion precipitation with 400 µL TM buffer (20 mM Tris, 5 mM MgCl_2_, pH 8.0). The nanostructures were characterized using agarose gel electrophoresis, TEM, UV spectroscopy, and DLS.

### Gel Preparation

First, 1 mL of solvent (such as H_2_O or chloroform) and a certain amount of gelator were added to the threaded bottle, heated at 100 °C, and stirred until the gelator was completely dissolved and cooled to RT. Then, the threaded bottle was inverted to observe and judge gel formation. The minimum amount of gelator required to form a gel was defined as the CGC. The gelator was dissolved in PEG200 and H_2_O (3:2, v/v), heated and dissolved in a reflux condenser, transferred to a threaded bottle, and cooled to RT to form blank ROS‐responsive supramolecular gels. The preparation of the ROS‐responsive supramolecular gels co‐loaded with ZnPCS_4_ and TGSAs was similar to that described above. The inclusions (ZnPCS_4_ and/or TGSAs) and gelator were dissolved in PEG200 and H_2_O (3:2, v/v), heated, and cooled to form the ZnPCS_4_‐loaded gel, TGSA‐loaded gel, and ZnPCS_4_‐TGSAs co‐loaded gel. The blank, ZnPCS_4_‐loaded, TGSAs‐loaded gel, and ZnPCS_4_‐TGSAs co‐loaded gel were identified as G1, G2, G3, and G4, respectively.

### Properties Characterization of Gel—Rheological Performance Assay

All experiments were conducted at 37 °C, and after lowering the roof to a clearance distance of 1 mm, excess hydrogel was scraped away. The hydrogel was balanced for 5 min before testing. The storage (G′) and loss (G′′) modulus of gels (G1, G2, G3, and G4) were examined by angular frequency sweep experiments from 0.1 to 100 rad s^−1^ at a fixed strain amplitude of 0.5%. Shear rate scans at 10.0 rad s^−1^ frequency were measured in the range of 0.1 to 100 s^−1^ to characterize the shear thinning behavior of different gels. All parameters were estimated using Advanced Rheology Expanded Systems (Malvern Kinexus lab+ system, UK).

### Properties Characterization of Gel—Evaluation of Gel Release In Vitro

Fluorescence spectra were used to characterize the drug‐release behavior of Cy5‐TGSAs loaded in gels in 0, 50, 150, and 250 mM H_2_O_2_. Gels loaded Cy5‐TGSAs (concentration of Cy5‐TGSAs = 1 µM) were placed in dialysis tubes containing 25 mL of the PBS buffer solution with different concentrations of H_2_O_2_. The dialysis tubes were gently incubated in a shaker (37 °C, 60 rpm) for 48 h. 1 mL release solution was taken out at predetermined intervals, and 1 mL fresh medium was added immediately. Released Cy5 was measured using fluorescence spectroscopy (F‐7100, Hitachi Co. Ltd., Japan), and the cumulative release of Cy5 at each time point was calculated (*n* = 3, mean ± SD).

### Photothermal Performance of TGSAs

PBS, Au NPs, and TGSAs were irradiated with L_2_ for 0, 3, 6, and 9 min. The temperature at each time point was recorded using a thermocouple. The experiment was repeated thrice.

### Cytotoxicity Assay

The biocompatibility and toxicity of the gelator were studied using the MTT and live/dead assays. First, phototoxicity was evaluated against L929 and 4T1 tumor cells. L929 and 4T1 cells were seeded in 96‐well plates and incubated for 24 h. The cells were irradiated at different laser wavelengths and times (L_1_/L_2_‐5 min, L_1_/L_2_‐10 min, L_1_+L_2_‐5 min, and L_1_+L_2_‐10 min) and incubated for 24 h. The medium was replaced with fresh medium containing MTT (0.5 mg mL^−1^, 100 µL) and continued incubation for 4 h. The medium was then replaced with DMSO, and the absorbance at 492 nm was measured using a microplate reader to evaluate cell viability. In addition, the biocompatibility of the gelator was assessed by culturing cells with different concentrations of the gelator extract. Different concentrations of gelators (1, 5, 10, 20, and 40 mg mL^−1^) were added to the medium. After 24 h, L929 and 4T1 cells were cultured in the filtered medium for 48 h, and cell viability was measured using the MTT assay.

The biocompatibility of the gel was studied using a live/dead assay. L929 and 4T1 cells were inoculated on a gel in a 24‐well plate and incubated for 24, 48, or 72 h. After different times, the cells were stained with Calcein/PI reagent (100 µL) for 20 min. CLSM was used to observe the growth and migration of cells inside the gel to assess its biocompatibility.

The cytotoxicity of TDNs was evaluated using an MTT assay. TDNs at different concentrations (10, 20, 50, 100, 200, and 500 nM) were incubated with L929 or 4T1 cells for 48 h, and cell viability was detected using the MTT assay.

### Production of ROS In Vitro

The production of ROS in vitro was measured via CLSM. 4T1 cells were inoculated in a glass substrate for 36 h, and then ZnPCS_4_ with different concentrations (0.1, 1, 10, 50, and 100 µM) were incubated with 4T1 cells for 4 h. The medium was then replaced with a medium containing DCFH‐DA. After 30 min, the cells were irradiated with L_1_. After rinsing with PBS, CLSM was used for capture.

### Cell Uptake

The internalization of TGSAs by normal and hypoxic cells was evaluated using CLSM and FCM. Normoxic and hypoxic 4T1 cells induced by CoCl_2_ were inoculated into glass culture dishes for 24 h. The medium was replaced with a medium containing Cy5‐TDNs, Cy5‐TGAs, or Cy5‐TGSAs for 1 and 3 h. The cells were fixed with 4% paraformaldehyde, stained with DAPI, and observed by CLSM. After trypsin digestion, PBS was used for resuspension, and FCM was used for quantitative detection of cell uptake.

### TGSAs Penetration In Vitro

MTSs, ideal models to simulate various physiological parameters of tumor tissue in vitro, were used to evaluate the permeability of TGSAs. 4T1 cells were inoculated into 96‐well plates containing 1% agarose gel and cultured at 37 °C for 3 days. When the diameter of MTSs reached nearly 200 µm, TGSAs were added and incubated for 6 h. After rinsing with PBS, CLSM imaging was used to observe and analyze the 3D Z‐stacking fluorescence intensity.

### Endolysosomal Escape

Hypoxic 4T1 cells were inoculated with the different formulations and cultured for 2 h. The culture medium was replaced with fresh medium, and the cells were incubated for 0, 1, and 4 h. The cells were stained with LysoTracker Green for 45 min and observed using CLSM.

### Photothermal Properties In Vitro

The 4T1 cells were inoculated and co‐cultured with TSAs and TGSAs for 4 h. After digestion; the cells were resuspended in a complete medium. The cells were irradiated with L_2_ and photographed using an NIR thermal imager at 0, 3, 6, and 9 min, and a photothermal curve was drawn.

### Expression of HSP90 In Vitro

4T1 cells were inoculated with a medium containing GA, TSAs, TGSAs, and TGSAs+L_2_ for 4 h. After digestion, the proteins were quantitatively detected using the BCA Protein Concentration Assay Kit (Solarbio). Proteins with different molecular weights were isolated and transferred to polyvinylidene fluoride (PVDF) membranes by 12% sodium dodecyl sulfate‐polyacrylamide gel electrophoresis (SDS‐PAGE). The proteins were incubated with Anti‐HSP90β antibody and Ultra Polymer Goat anti‐Mouse IgG (H&L)‐HRP, respectively, and imaged on the Chemi Doc XRS + system (Bio‐Rad, USA).

### Evaluation of Antitumor Efficiency In Vitro

Hypoxic 4T1 cells were inoculated in a medium containing Free GA, TGAs, TGSAs, P/TGSAs (ZnPCS_4_/TGSAs) +L_1_, P/TGSAs+L_2_, P/TSAs (ZnPCS_4_/TSAs) +L_1_+L_2_, or P/TGSAs+L_1_+L_2_. (+L_1_ or+L_2_ represents administration of the L_1_ or L_2_ laser). The MTT assay was used to evaluate cell viability.

### Cell Apoptosis Assay

Hypoxic 4T1 cells were inoculated in a medium containing Free GA, TGAs, TGSAs, P/TGSAs (ZnPCS_4_/TGSAs) +L_1_, P/TGSAs+L_2_, P/TSAs (ZnPCS_4_/TSAs) +L_1_+L_2_, or P/TGSAs+L_1_+L_2_. (+L_1_ or+L_2_ represents administration of the L_1_ or L_2_ laser). After 12 h, the cells were digested, centrifuged, and treated according to the procedures described in the Annexin V‐FITC Apoptosis Detection Kit (Beyotime). Apoptosis was analyzed by FCM.

### Proteomics Analysis

After incubation with P/TGSAs for 24 h, the 4T1 cells were exposed to L_1_ and L_2_ for 5 min. Untreated cells were used as controls, and each experiment was repeated three times (*n* = 3). Cells from each group were digested, centrifuged, and collected after repeated cleaning with PBS and stored in a −80 °C environment. The collected cells were cleaved using a cleavage buffer (1% SDS and 1% protease inhibitor cocktail), and the collected proteins were determined using a BCA kit. Equal amounts of proteins from each group were extracted and cleaved. The collected peptides were analyzed by liquid chromatography‐mass spectrometry (LC‐MS) and ionized using a timsTOF Pro 2 mass spectrometer for data collection. The experiment retrieved DIA data using the search engine DIA‐NN (v1.8) and default software parameters. The Mus_musculus_10090_SP_20230103 database was used. PCA was used for statistical data analysis, and GO analysis, COG/KOG, and KEGG approaches were used for the functional classification of differentially expressed proteins.

### Antitumor Efficiency In Vivo

All animal experiments were approved by the Animal Experiment Ethics Committee of Qingdao University (QDU‐AEC‐2023086). Tumor‐bearing BALB/c mice were randomly divided into seven groups: Saline, gel, G@P/TGSAs, G@P/TGSAs+L_1_, G@P/TGSAs+L_2_, G@P/TSAs +L_1_+L_2_, and G@P/TGSAs+L_1_+L_2_. When the tumor volume reached 100–150 mm^3^, the drug was administered by in situ injection (ZnPCS_4_:50 µg/mL, TGSAs: 1 µM). The tumor was irradiated with L_1_ (5 min) after injection to promote gel degradation. After 6 h, L_2_ was irradiated, and the drug was administered once every 3 days for a total of four times. The body weight and tumor volume of the mice were measured every 3 days (*V* = tumor length tumor width ^2^/ 2).

After 19 days, 4T1 tumor‐bearing mice were randomly selected and sacrificed, and the tumor tissues and major organs (heart, liver, spleen, lungs, and kidneys) were collected. At the same time, blood samples of the mice were collected for blood indicator tests. After 4% paraformaldehyde fixation, the tumor tissues were stained with H&E, Ki‐67, TUNEL, and HSP90 staining to further evaluate the biosecurity and therapeutic effect in vivo, and the survival rate of the remaining mice was calculated within 60 days. The biosecurity of the drug delivery system was evaluated by H&E staining of major organs and detection of blood biochemical indices.

### Penetration of TGSAs In Vivo

Cy5 and Cy5‐TGSAs were injected into the tumor sites to verify deep penetration at tumor sites. 12 h after injection, the tumor tissue was frozen, and the fluorescence intensity of Cy5 in the tumor tissues was analyzed using CLSM imaging.

### Generation of ROS and PTT Effect In Vivo

ROS generation at the tumor site was verified using SOSG as a fluorescent probe. Briefly, G@P/TGSAs were injected into tumor sites and exposed to L_1_ for 5 min. After 6 h, the mice were sacrificed, and the tumors were collected for frozen sections. CLSM imaging was performed to evaluate the effects of ROS generation in vivo. A near‐infrared thermal imager was used to monitor changes in tumor temperature during L_2_ irradiation to evaluate the effect of PTT in vivo. Saline, G@P/TSAs, or G@P/TGSAs were injected into the tumor sites. Temperature changes in the tumors were recorded using a thermal imaging camera.

### In Vivo Imaging

The distribution degradation of G@P/TGSAs in mice was verified using an IVIS Spectrum (PerkinElmer, USA). Cy5 and G@P/Cy5‐TGSAs were injected into the tumor, the mice were anesthetized with isoflurane, and the fluorescent signal of Cy5 was detected. Images of the mice were taken at 1, 6, 12, 24, and 48 h after injection. After 48 h, major organs and tumors were collected for in vitro imaging.

### Statistical Analysis

Data were expressed as mean ± SD and either the Student's *t*‐test or one‐way ANOVA were used (*p* > 0.05: n.s., *p < 0.05, **p < 0.01 and ***p < 0.001).

## Conflict of Interest

The authors declare no conflict of interest.

## Author Contributions

F.L. and J.Y. equally contributed to this work. Y.L. conceived this study. F.L. and J.Y. developed the most experiments and analyzed figures. F.L. and Y.L. wrote the manuscript. L.X. and X.T. participated in the synthesis and simulation of materials. C.W., Y.Z., and J.C. participated in the animal experiments. Y.S. and B.H. reviewed and revised the manuscript. All authors review and agree to approve the final manuscript.

## Supporting information

Supporting Information

## Data Availability

The data that support the findings of this study are available from the corresponding author upon reasonable request.
