# Corrigendum to ““Cicada Out of the Shell” Deep Penetration and Blockage of the HSP90 Pathway by ROS‐Responsive Supramolecular Gels to Augment Trimodal Synergistic Therapy”

**DOI:** 10.1002/advs.202512868

**Published:** 2025-07-25

Adv. Sci. 2024, 11, 2401214.

https://doi.org/10.1002/advs.202401214; Adv. Sci. 2024, 11, 2401214

Histological analyses of heart toxicity through hematoxylin‐eosin (H&E) staining at row one in Figure 8A. The photo of Group 3 was misplaced due to carelessness, resulting in a duplication with Group 1. Please find the picture with the correct photo below.


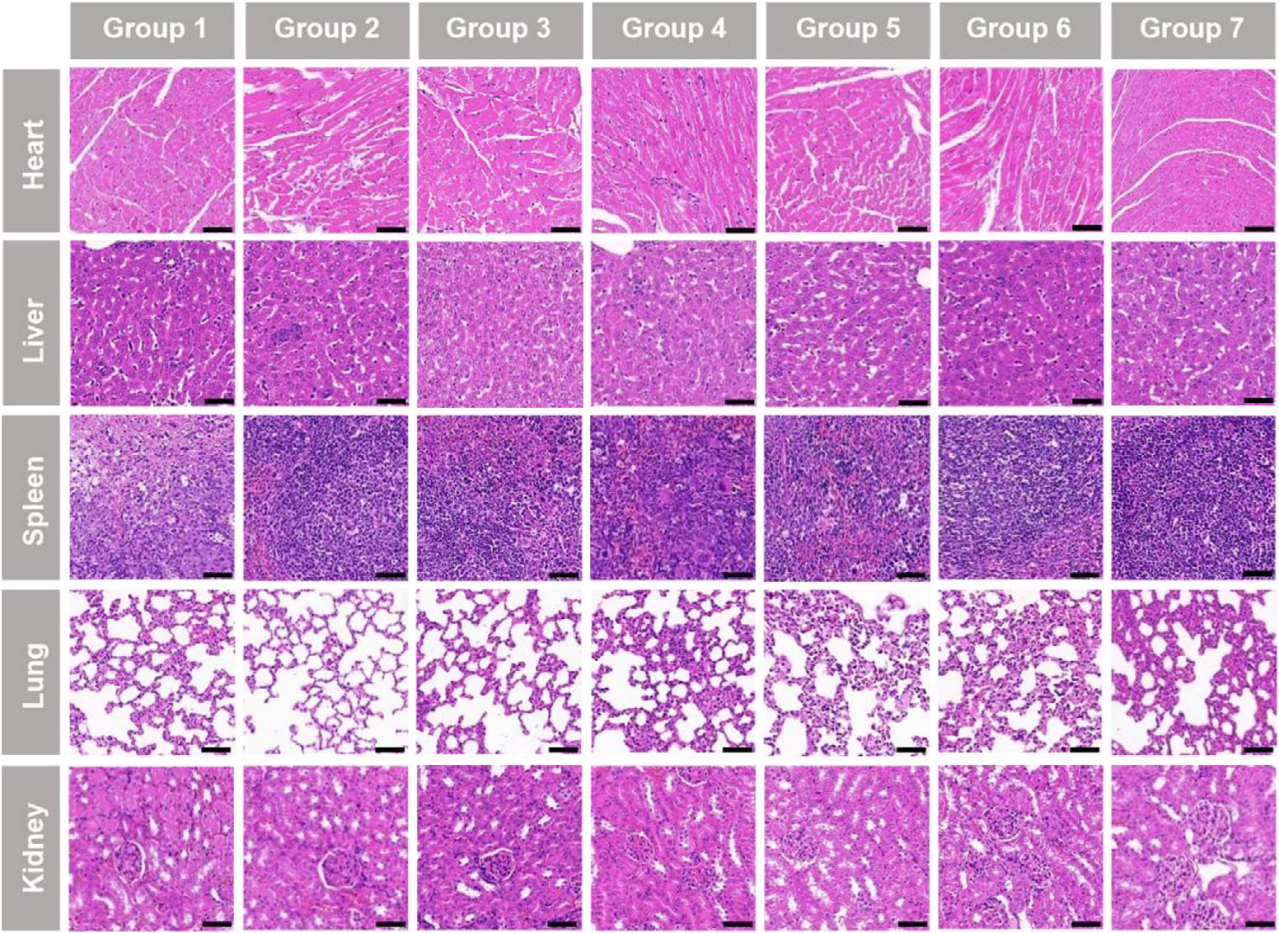


We apologize for this error.

