# Supplementary material for: “Cicada Out of the Shell” Deep Penetration and Blockage of the HSP90 Pathway by ROS‐Responsive Supramolecular Gels to Augment Trimodal Synergistic Therapy
Source: Adv Sci (Weinh). 2024 Apr 22;11(25):2401214. doi: 10.1002/advs.202401214 (PMC11220648; doi:10.1002/advs.202401214)
Supplement: Supplementary file 1 — Supporting Information [file ADVS-11-2401214-s001.docx]

Supporting Information

"Cicada Out of the Shell" Deep penetration and blockage of HSP90 pathway by ROS-responsive supramolecular gels to augment trimodal synergistic therapy

*Fashun Li, Jianqin Yan, Chen Wei, Yi Zhao, Xiaowen Tang, Long Xu, Bin He, Yong Sun*, Jing Chang, Yan Liang**

**1.1 Synthesis of 11****-mercaptoundecanoic acid**

11-bromoundecanoic acid (1.1 g, 5 mmol, 1 eq), thioureide (0.57 g, 7.5 mmol, 1.5 eq), and anhydrous ethanol (15 mL) were placed in 100 mL round-bottom flask, heated at 100℃ for reflux for 24 h, and the solvent was removed by rotary evaporator and added to excess sodium hydroxide solution. The solution was stirred at high temperature for 20 h, and the reaction system was protected by N_2_. The pH was adjusted by sulfuric acid solution to acidity under low temperature. The mixed solution was extracted repeatedly with methylene chloride, dried with anhydrous sodium sulfate, and the product 11-mercaptoundecanoic acid (1.23 g, 84%) was obtained after removing the solvent.

**1.2 Synthesis of Thioketal (TK)**

11-mercaptoundecanoic acid (11 g, 50 mmol), acetone (2.90 g, 49.1 mmol) and catalyzed amounts of trifluoroacetic acid (TFA) were placed in a round-bottled flask and stirred in RT for 6 h. After the reaction, the mixture was placed in an ice bath until crystallization was complete. The solution is then filtered and washed with hexane and cold water. A white product (TK) was obtained after drying overnight in vacuum drying (18.01 g, 79% yield).

**1.3 Synthesis conjugate of gelator precursor (Phe-TK-Phe)**

First, triethylamine (3.70 mL, 26.4 mmol) was used to remove the acid molecules contained in L-phenylalanine methyl ester salt (4.30 g, 22 mmol). TK (2.52 g, 10 mmol) and CDI (3.57 g, 22 mmol) were dissolved in anhydrous dichloromethane (25 mL) for activation. The L-phenylalanine methyl ester solution was mixed with the above solution and stirred for 24 h under the condition of N_2_. After the solvent was removed, the dissolved product of tetrahydrofuran was added, filtered, concentrated and purified to obtain Phe-TK-Phe (4.82 g, 84.1% yield).

**1.4 Synthesis of ROS-responsive gelator**

Phe-TK-Phe (5.74 g, 10 mmol) was dissolved in mixture solvent (30 mL) of dichloromethane and methanol (v: v = 1: 2) under N_2_. Subsequently, hydrazine hydrate (2 mL) was injected, and the mixed solution was stirred for 24 h under ice bath conditions. The mixture was filtered and the solid was washed by dichloromethane several times. The solid was collected and vacuum-dried to obtain the gelator (2.58 g, yield, 90.1%).

**1.5 Verification of ROS-responsiveness of gelator**

The ROS response of gel was characterized by ^1^H NMR spectroscopy to study the structural changes of gel treated with ROS. H_2_O_2_ (0.2 mL) was added to PBS (9.8 mL) to obtain 200 mM H_2_O_2_ solution. Gelator (20 mg) was dissolved in 5 mL of acetone and then mixed with the above solution. After it was incubated at 37 ℃ for 24 h, the mixed solution was lyophilized, and the lyophilized powder was characterized by ^1^H NMR.

**1.6 Characterization of microstructure of gel**

The microstructure of the gel was observed by scanning electron microscope (SEM). The gel was prepared by adding a certain amount of gelator to chloroform or ethanol. The gel was prepared into a xerogel and placed on carbon conductive tape. The microstructure of the xerogel was observed by SEM after gold sputtering.

**1.7 Gelation mechanism and** **molecular dynamics of gelator**

The self-assembly mechanism was studied ^1^H NMR and FT-IR. ^1^H NMR: The gel concentrations of 10, 20, and 25 mg/mL were prepared by adding gelator to chloroform-d. FT-IR: Chloroform gel with the concentration of 10, 25 mg/mL was prepared by gelator. Small amounts of gelator/xerogel and potassium bromide were ground into powder and pressed into flakes for FT-IR.

The gel driving force and properties of the gelator were further verified by using computer molecular dynamics simulations of the structural stability and intermolecular forces of the gelators, which were carried out in the software Gromacs-4.6.7. Here, the structure of ROS-responsive gelator was constructed and placed in the middle of the simulation box. The simulation box will perform MD simulation of 200 ns NPS at a pressure of 1 bar (P) and a temperature of 300 K (T), keeping the step size at 1fs. Subsequently, the g-mmpbsa tool was used to calculate the free energy of the gelator simulation structure.

**
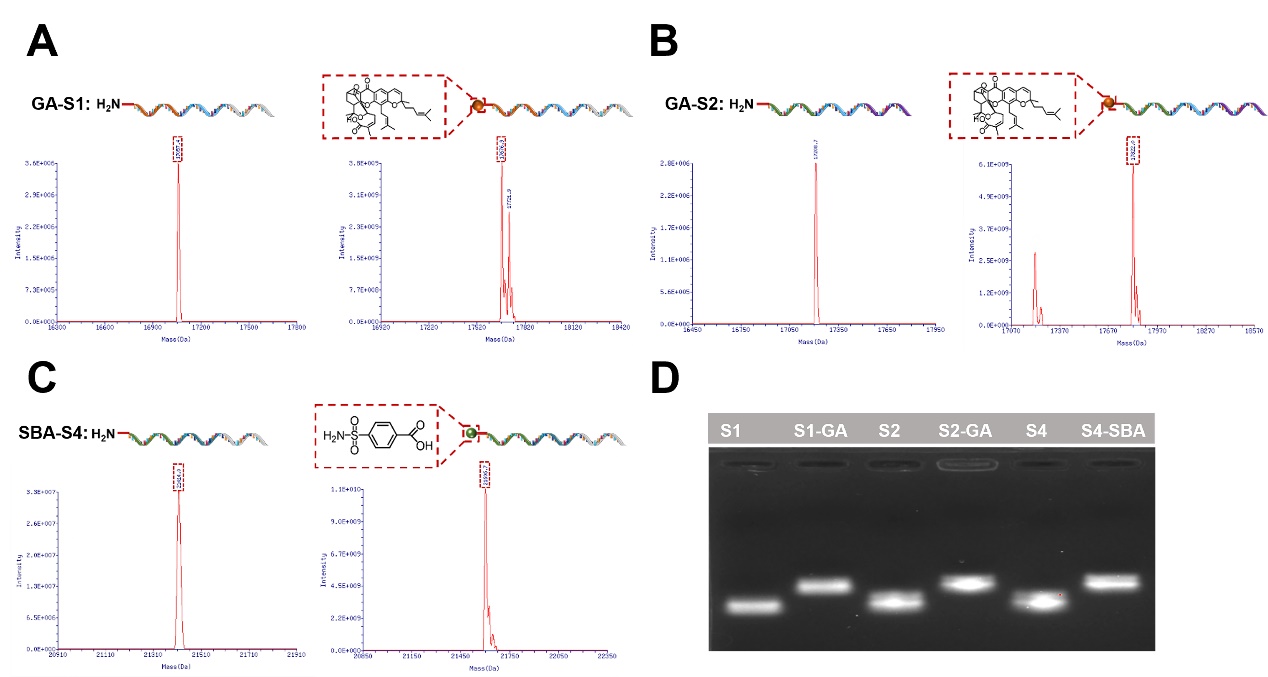
**

**Figure S1.** (A), (B) and (C) showed the ESI-MS before and after modification of DNA oligonucleotide. (D) The image of oligonucleotide gel electrophoresis before and after modification.


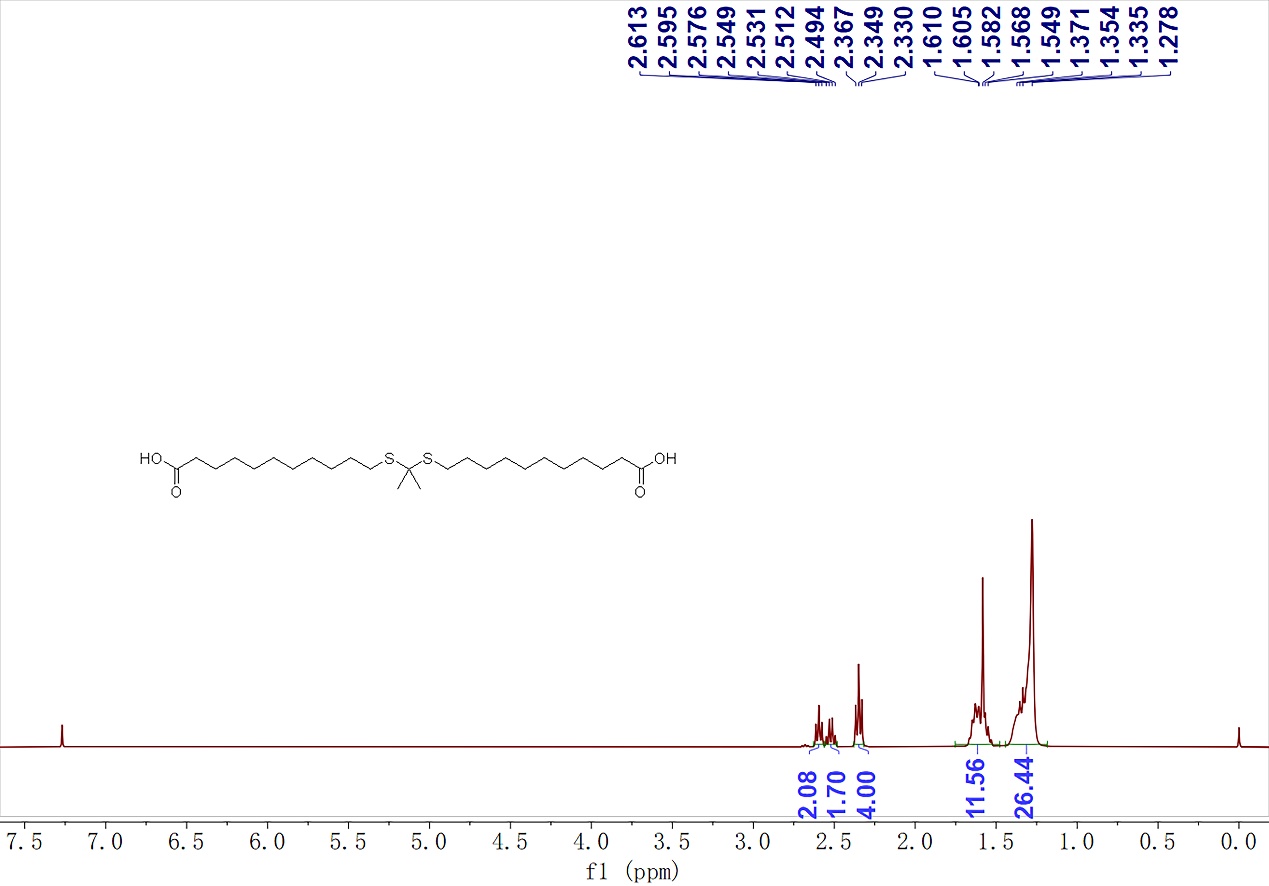


**Figure S2.** ^1^H NMR spectra of TK

**
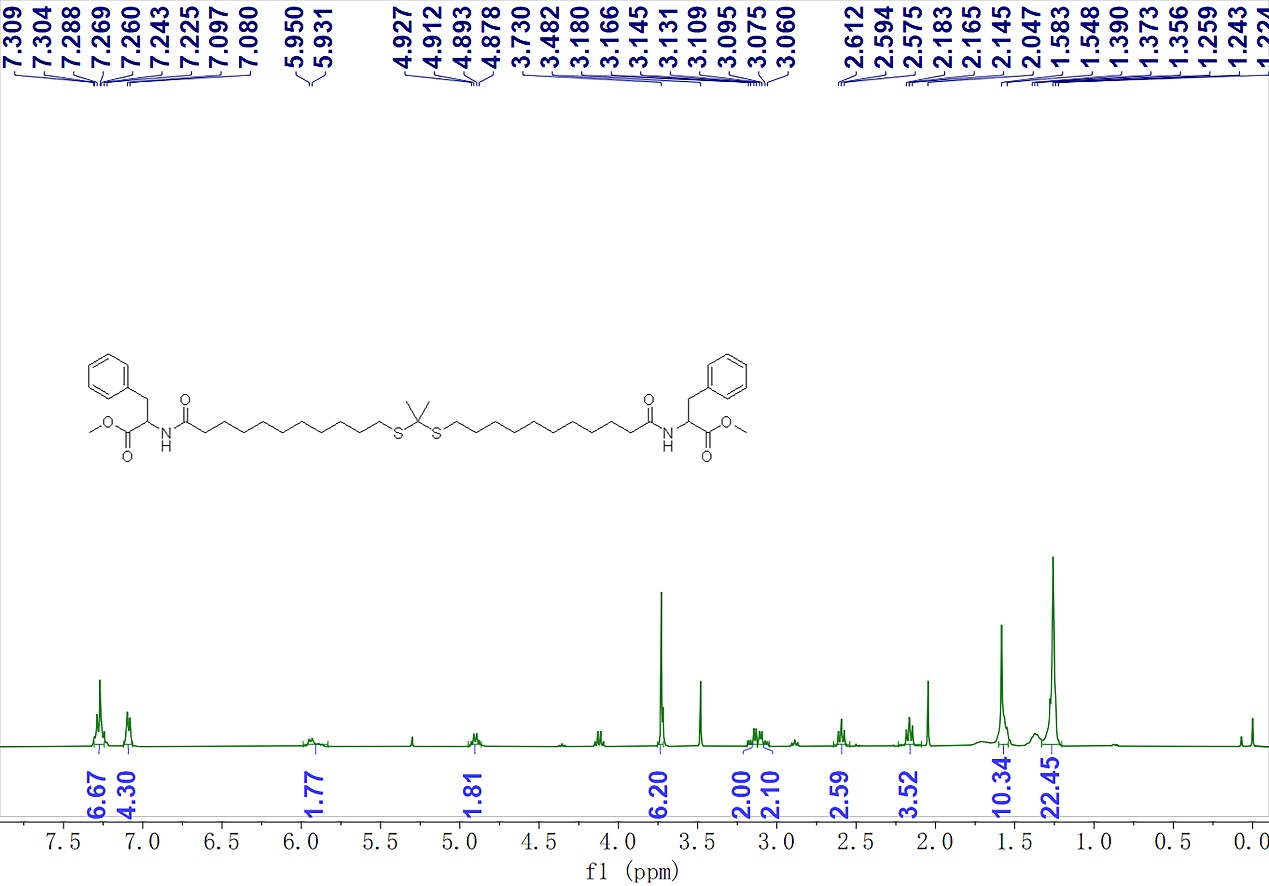
**

**Figure S3.** ^1^H NMR spectra of Phe-TK-Phe


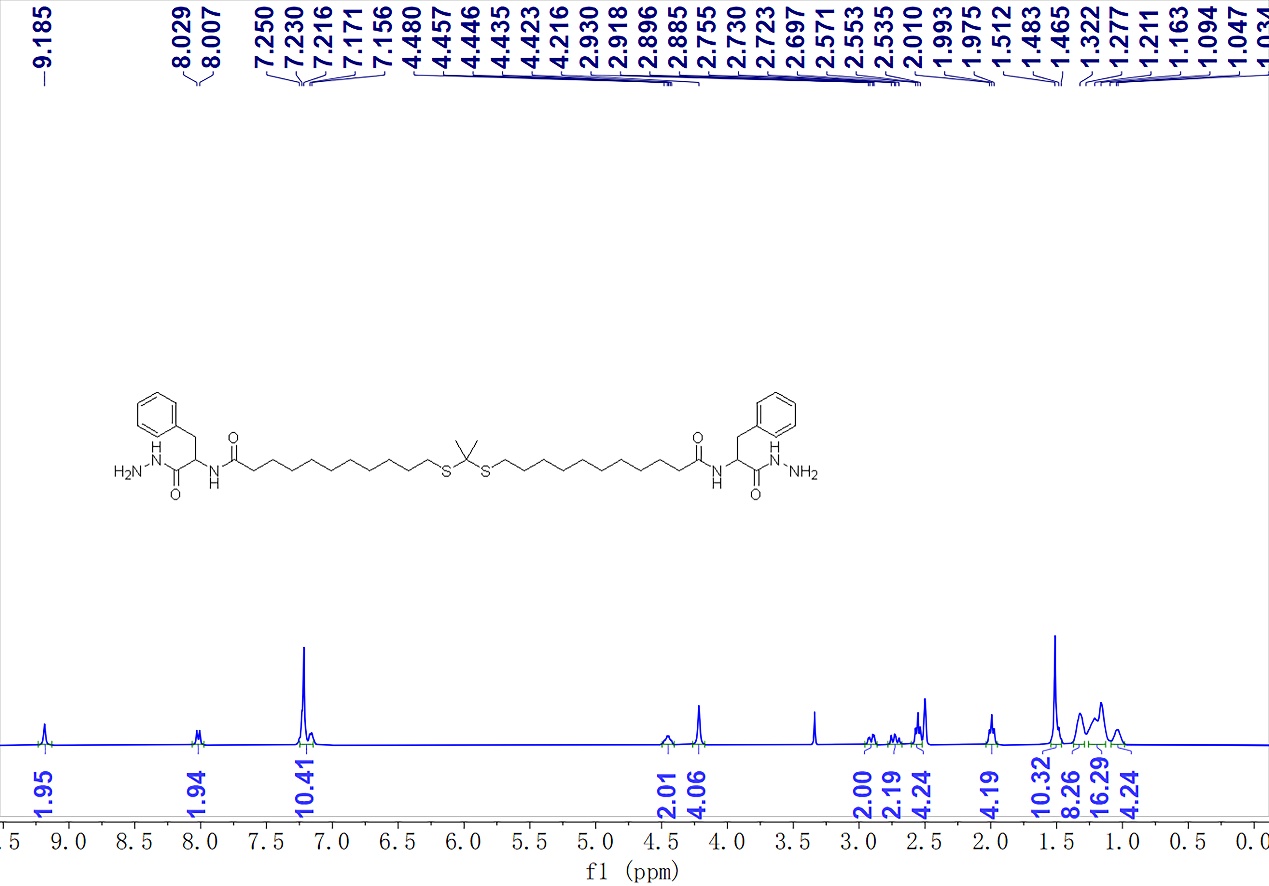


**Figure S4.** ^1^H NMR spectra of gelator


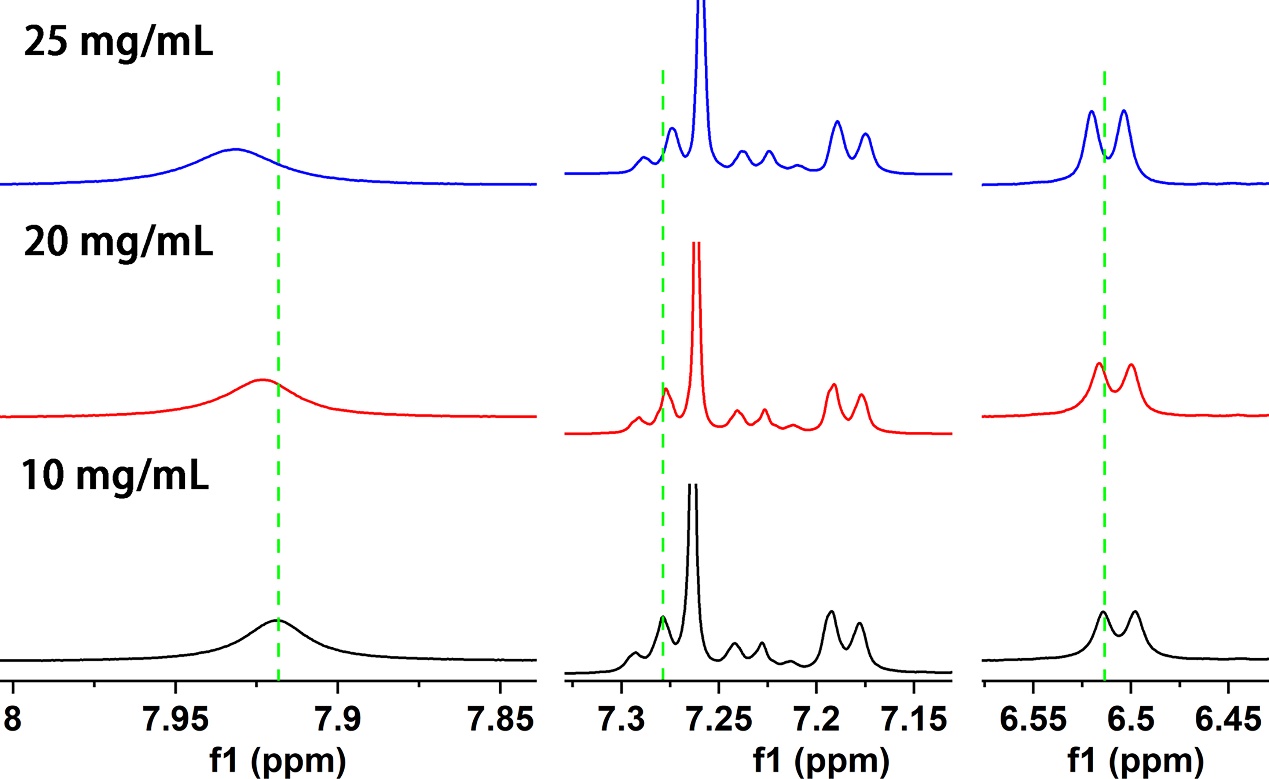


**Figure S5.** ^1^H NMR spectra of different concentrations of gelator
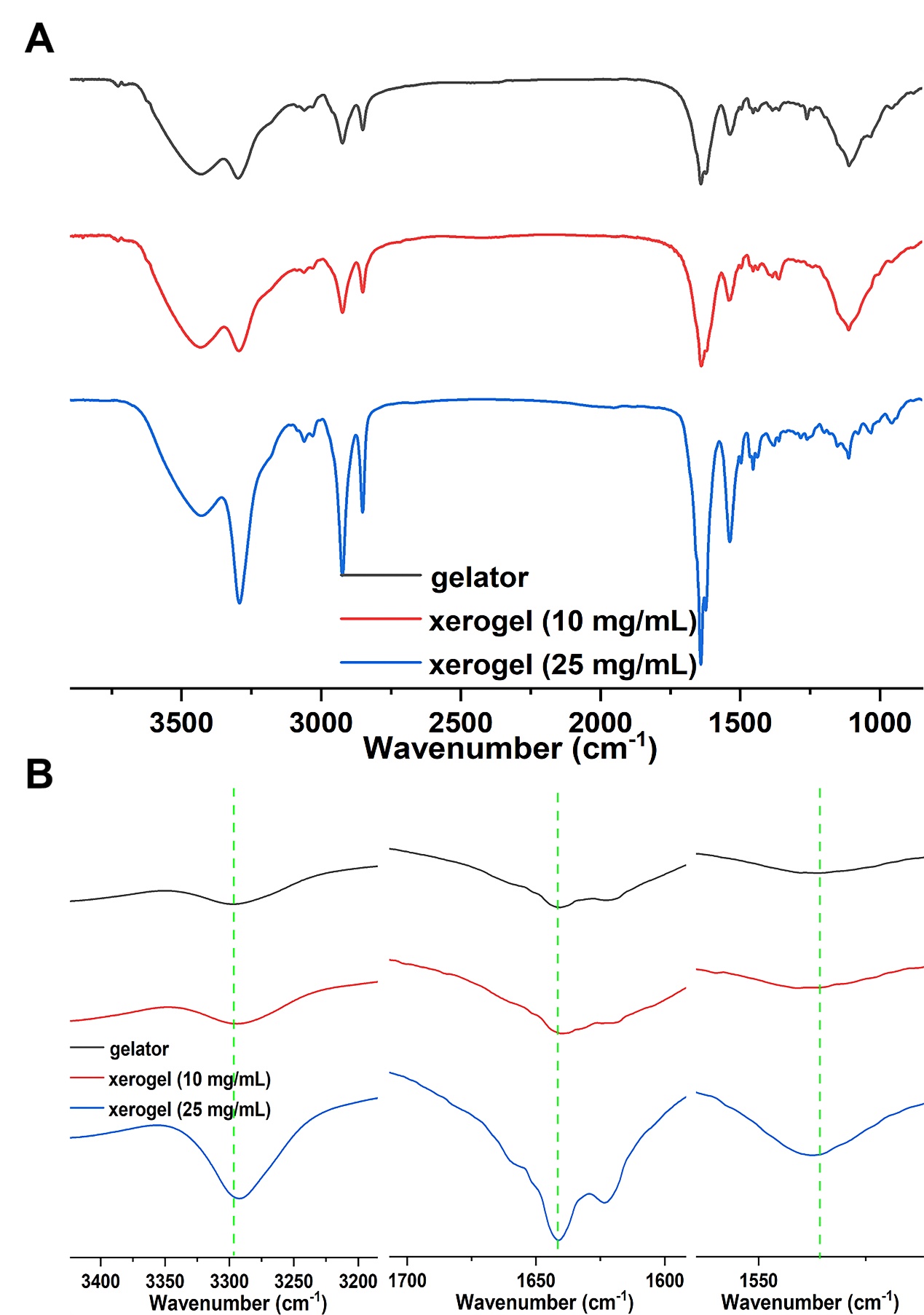


**Figure S6.** (A) FT-IR of gelator and xerogel; (B) Enlarged FT-IR of gelator and xerogel

**
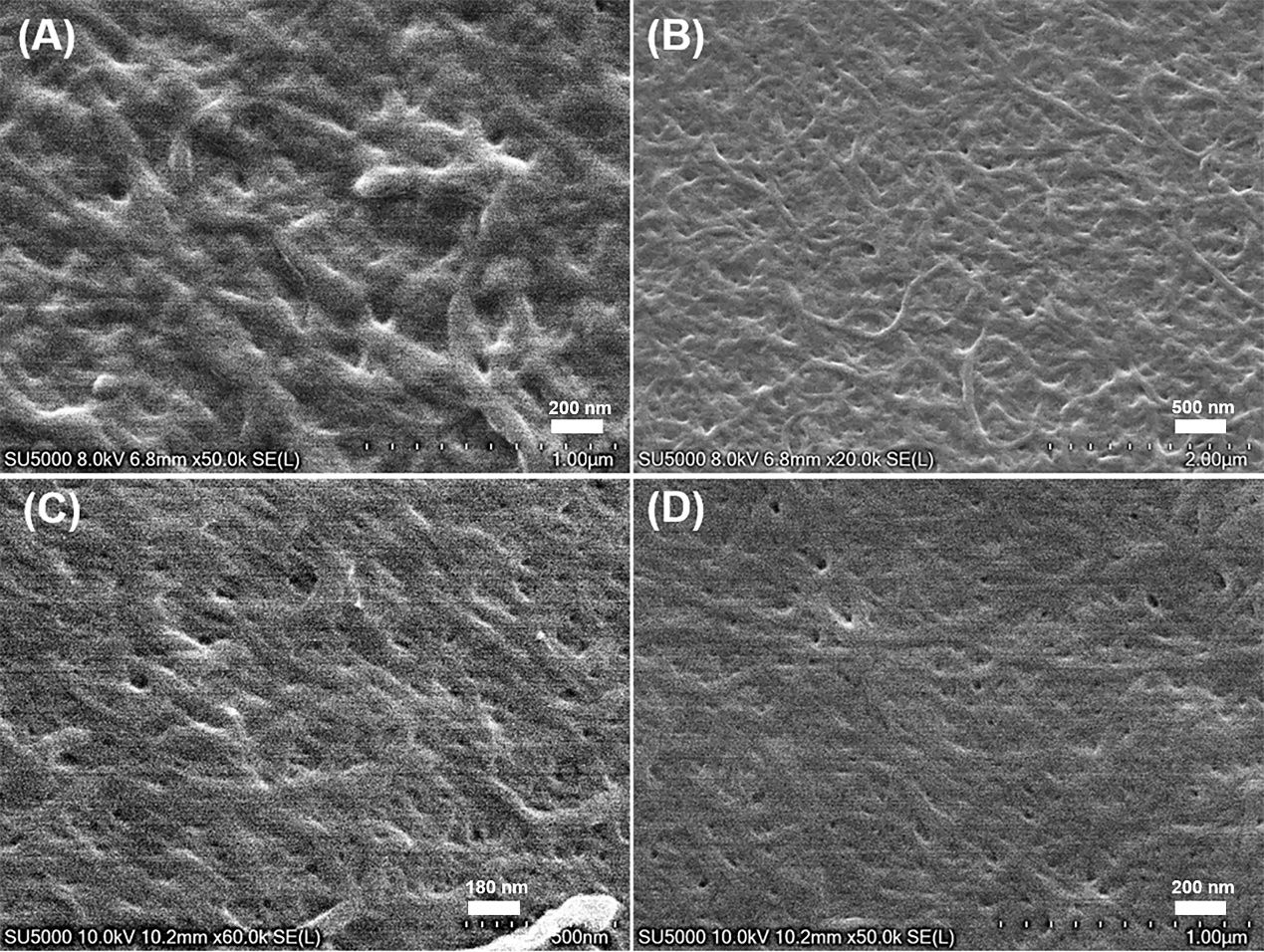
**

**Figure S7.** SEM images of xerogel. (A) and (B) were chloroform gel of gelator 3 with a concentration of 10 mg/mL and 15 mg/mL; (C) and (D) were ethanol gel of gelator 3 with a concentration of 10 mg/mL and 15 mg/mL, respectively

**
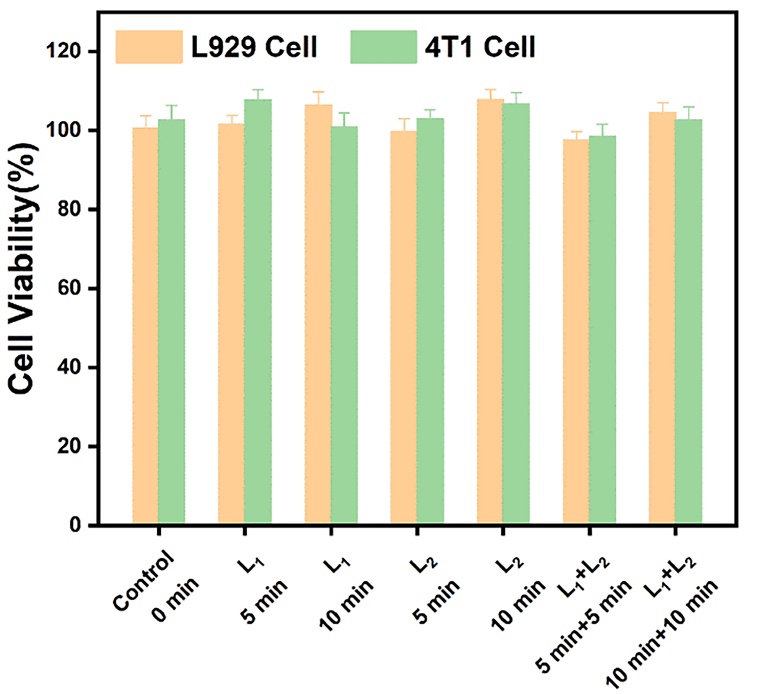
**

**Figure S8.** Cytotoxicity of L929 and 4T1 with two types of near-infrared laser

**
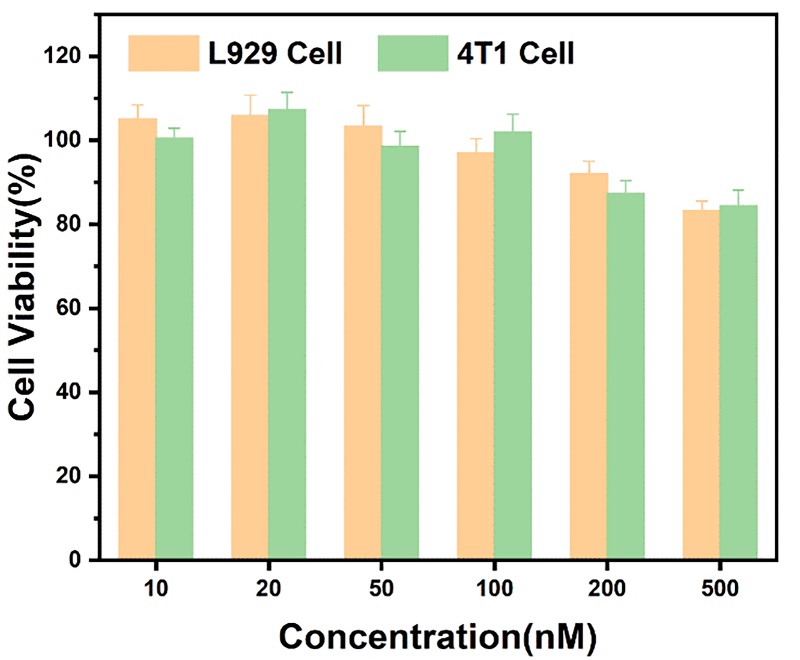
**

**Figure S9.** Cytotoxicity of TDNs incubated L929 and 4T1 cells at different concentrations

**
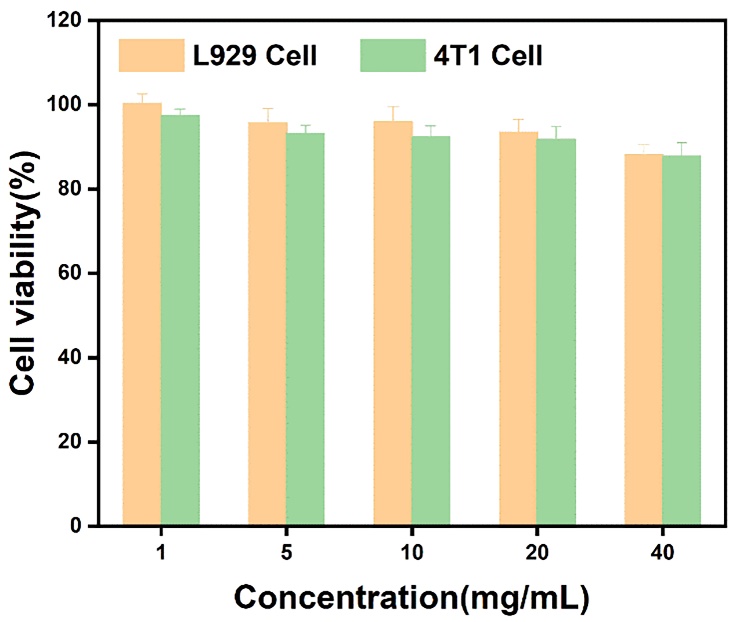
**

**Figure S10.** Cytotoxicity of gel-incubated L929 and 4T1 cells at different concentrations

**
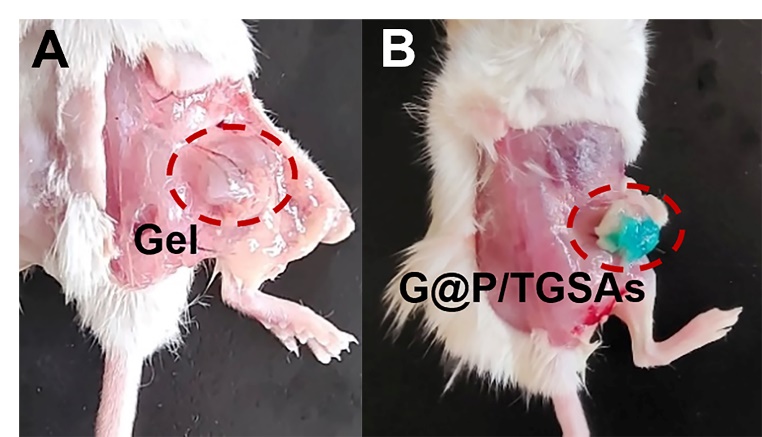
**

**Figure S11.** Pictures G1 and G4 in gelatinized state after injection into tumor in mice

**
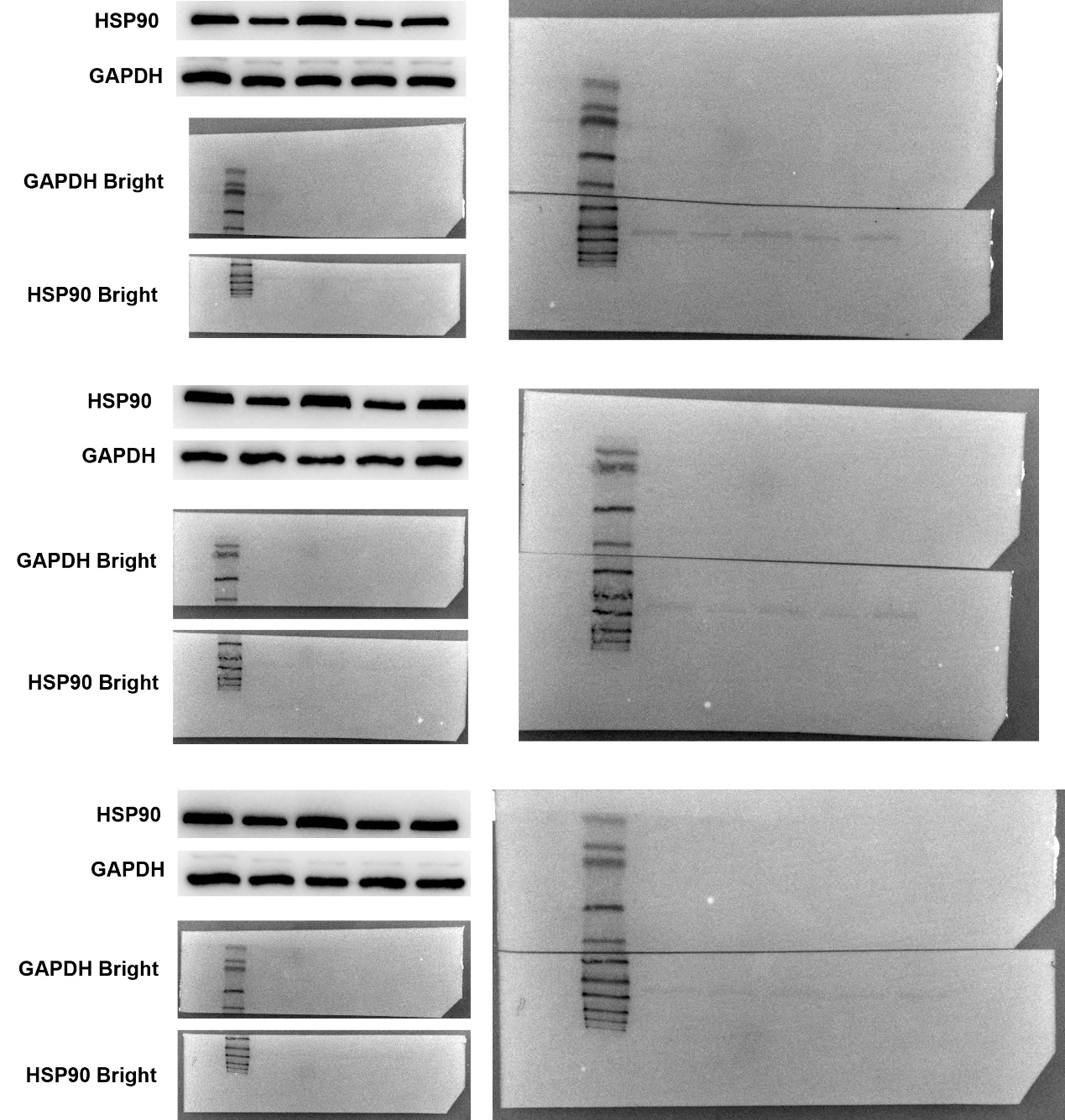
**

**Figure S12.** Original images of WB

**Table S1**. The modified DNA single strands were used in this study

| **ssDNA** | **Sequence (5**′**- 3**′**)** |
| --- | --- |
| **S1** | ACATTCCTAAGTCTGAAACATTACAGCTTGCTACACGAGAAGAGCCGCCATAGTA |
| **S2** | TATCACCAGGCAGTTGACAGTGTAGCAAGCTGTAATAGATGCGAGGGTCCAATAC |
| **S3** | TCAACTGCCTGGTGATAAAACGACACTACGTGGGAATCTACTATGGCGGCTCTTC |
| **S4** | TTCAGACTTAGGAATGTGCTTCCCACGTAGTGTCGTTTGTATTGG ACCCTCGCAT |
| **NH_2_-C6-S1** | NH2-C6-  ACATTCCTAAGTCTGAAACATTACAGCTTGCTACACGAGAAGAGCCGCCATAGTA |
| **NH_2_-C6-S2** | NH_2_-C6-  TATCACCAGGCAGTTGACAGTGTAGCAAGCTGTAATAGATGCGAGGGTCCAATAC |
| **SH-C6-S3** | SH-C6-S3  TCAACTGCCTGGTGATAAAACGACACTACGTGGGAATCTACTATGGCGGCTCTTC |
| **NH_2_-C6-S4** | TTCAGACTTAGGAATGTGCTTCCCACGTAGTGTCGTTTGTATTGG ACCCTCGCAT |
| **Cy5-S2** | Cy5- TATCACCAGGCAGTTGACAGTGTAGCAAGCTGTAATAGATGCGAGGGTCCAATAC |

**Table S2.** Gelation of gelator in different solvents and critical gel Concentration (CGC)

| Entry | Organic solvent | Gelator 3 CGC at 25℃ (mg/mL) |
| --- | --- | --- |
| 1  2  3  4  5  6  7  8  9  10  11  12  13  14  15  16  17  18 | Petroleum ether  Cyclohexane  Diethyl ether  Ethyl acetate  Dichloromethane  Chloroform  Acetone  Acetonitrile  Methanol  Ethanol  Ethylene glycol  Dimethyl sulfoxide  PEG200  PEG400  H_2_O  Ethanol : H_2_O (1:1)  PEG200 : H_2_O (1:1)  PEG200 : H_2_O (3:2) | I  I  I  G (5 mg/mL)  G (3 mg/mL)  G (10 mg/mL)  S  G (2 mg/mL)  G (7 mg/mL)  G (6 mg/mL)  G (12 mg/mL)  S  G (15 mg/mL)  P  P  G (2 mg/mL)  G (18 mg/mL)  G (13 mg/mL) |

a) I: insoluble, the gelator is completely insoluble in the solvent;

b) G: gel, the gelator is able to gelation the solvent;

c) S: solution, the gelator is completely soluble in the solvent;

d) P: precipitate, the gelator dissolves after heating, and precipitates after cooling to room temperature.

**Table S3.** Serum biochemistry data of white blood cell count (WBC), red blood cell count (RBC), blood platelet count (PLT), C-reactive protein (CRP) and neutrophil granulocyte (GR#). (n=5)

| Samples | WBC (10^-3^/μL) | RBC (10^6^/μL) | PLT (10^6^/μL) | CRP (mg/L) | GR# (10^-3^/μL) |
| --- | --- | --- | --- | --- | --- |
| 1 | 7.95±0.58 | 6.34±0.75 | 394.0±9.6 | 3.85±0.34 | 1.63±0.12 |
| 2 | 7.93±0.79 | 5.35±0.45 | 445.0±9.2 | 3.92±0.31 | 1.69±0.13 |
| 3 | 7.96±0.25 | 6.08±0.45 | 399.0±9.5 | 3.97±0.32 | 1.67±0.23 |
| 4 | 7.95±4.28 | 6.34±0.38 | 397.0±9.2 | 4.03±0.39 | 1.58±0.24 |
| 5 | 6.92±0.67 | 5.78±0.46 | 390.0±7.7 | 3.95±0.32 | 1.64±0.18 |
| 6 | 7.04±0.74 | 5.87±0.62 | 399.0±6.9 | 3.86±0.34 | 1.59±0.25 |
| 7 | 7.99±0.17 | 5.85±0.59 | 396.0±7.4 | 3.82±0.25 | 1.68±0.24 |
| 8 | 6.84±0.26 | 5.95±0.79 | 393.0±7.8 | 3.79±0.36 | 1.61±0.19 |
| Normal range | 2.3~31.6 | 2.2~15 | 270~1100 | 0~7 | 1.5~2.0 |

1: Normal; 2: Control; 3: Gel; 4: G@P/TGSAs; 5: G@P/TGSAs+L_1_; 6: G@P/TGSAs+L_2_；7：G@P/TSAs+L_1_+L_2_; 8: G@P/TGSAs+L_1_+L_2_
